# Differential transcriptome analysis reveals insight into monosymmetric corolla development of the crucifer *Iberis amara*

**DOI:** 10.1186/s12870-014-0285-4

**Published:** 2014-11-19

**Authors:** Andrea Busch, Stefanie Horn, Sabine Zachgo

**Affiliations:** Department of Botany, Osnabrück University, Barbarastrasse, 11, Osnabrück, 49076 Germany

**Keywords:** Brassicaceae, Monosymmetry, *CYC*, *TCP1*, RNA-Seq, Microarray

## Abstract

**Background:**

In the co-evolution between insects and plants, the establishment of floral monosymmetry was an important step in angiosperm development as it facilitated the interaction with insect pollinators and, by that, likely enhanced angiosperm diversification. In *Antirrhinum majus*, the *TCP* transcription factor *CYCLOIDEA* is the molecular key regulator driving the formation of floral monosymmetry. Although most Brassicaceae form a polysymmetric corolla, six genera develop monosymmetric flowers with two petal pairs of unequal size. In the monosymmetric crucifer *Iberis amara,* formation of the different petal pairs coincides with a stronger expression of the *CYC*-homolog *IaTCP1* in the small, adaxial petals.

**Results:**

In this study, RNA-Seq was employed to reconstruct the petal transcriptome of the non-model species *Iberis amara*. About 9 Gb of sequence data was generated, processed and re-assembled into 18,139 likely *Iberis* unigenes, from which 15,983 showed high sequence homology to *Arabidopsis* proteins. The transcriptome gives detailed insight into the molecular mechanisms governing late petal development. In addition, it was used as a scaffold to detect genes differentially expressed between the small, adaxial and the large, abaxial petals in order to understand the molecular mechanisms driving unequal petal growth. Far more genes are expressed in adaxial compared to abaxial petals implying that *IaTCP1* activates more genes than it represses. Amongst all genes upregulated in adaxial petals, a significantly enhanced proportion is associated with cell wall modification and cell-cell signalling processes. Furthermore, microarrays were used to detect and compare quantitative differences in TCP target genes in transgenic *Arabidopsis* plants ectopically expressing different TCP transcription factors.

**Conclusions:**

The increased occurrences of genes implicated in cell wall modification and signalling implies that unequal petal growth is achieved through an earlier stop of the cell proliferation phase in the small, adaxial petals, followed by the onset of cell expansion. This process, which forms the monosymmetric corolla of *Iberis amara*, is likely driven by the enhanced activity of *IaTCP1* in adaxial petals.

**Electronic supplementary material:**

The online version of this article (doi:10.1186/s12870-014-0285-4) contains supplementary material, which is available to authorized users.

## Background

In the co-evolution between plants and insects, the development of floral monosymmetry was an important step, facilitating angiosperm speciation as a response to the adaptation to the visual senses of insect pollinators [1]. Monosymmetry, thus, likely functioned as a morphological key innovation and evolved independently in different angiosperm lineages [2,3].

Groundbreaking research almost two decades ago identified the *TCP* transcription factor *CYCLOIDEA (CYC)* as the molecular key regulator of monosymmetry development in *Antirrhinum* [4]. *CYC* and its paralog *DICHOTOMA* are expressed in the adaxial part of developing *Antirrhinum* flowers, where they guide the acquisition of adaxial identities of second and third whorl organs [4,5]. *CYC* belongs to the *CYC2* clade of the TCP transcription factor family [6] and in all core eudicot species analysed so far, monosymmetry development is controlled by *CYC2* clade genes (e.g. [7-11]).

The majority of crucifers (Brassicaceae) develop a polysymmetric corolla and only six genera form flowers with two petal pairs of different sizes [12]. In *Iberis amara,* unequal petal pair formation correlates with a stronger expression of the *CYC2* clade gene *IaTCP1* in the smaller, adaxial petals. Comparison of adaxial and abaxial epidermal cell sizes revealed that petal size differences are due to a differential rate of cell proliferation [10]. In a *peloric* flower variant, forming only large, abaxialized petals, the *IaTCP1* expression is dramatically decreased. Transgenic *Arabidopsis* plants overexpressing the cruciferous *CYC2* transcription factors *IaTCP1* from *Iberis amara* and *TCP1* from *Arabidopsis,* both, produce similar flowers with smaller petals. For plants overexpressing *IaTCP1* this was shown to be due to a reduction in cell number [10]. Contrarily, ectopic expression of *CYC* from *Antirrhinum* results in transgenic plants forming flowers with larger petals, a consequence of an increase in cell size [13]. This demonstrates that the function of the two crucifer proteins is mainly conserved, whereas that of CYC from the more distantly related species *Antirrhinum* likely diverged [10]. *Iberis* petals are initiated simultaneously as little bulges and the onset of an unequal size development can be detected around the start of stamen differentiation. From this point on, adaxial and abaxial petals develop differentially throughout flower development. The major difference in petal size, however, is acquired during late flower development, when a size difference of 1.6-fold, just after anthesis (stage A1) increases to 3.7-fold in fully mature flowers (stage A2) [10].

This raises the question about the molecular network that realises differential petal growth. Comprehensive research has been conducted analysing the genetic basis of general floral organ size determination, which is regulated through several independent pathways (reviewed in [14,15]). Initial petal growth is achieved through cell proliferation that is later maintained only in restricted regions [14]. Growth via cell division ceases and petals acquire their final size through cell elongation, a transition that seems to occur during later stages of flower development, after the maturation of microspores [16-19]. The switch to cell elongation goes along with an increased expression of cell wall synthesis and cell wall metabolization genes [20].

Thus, important determinators of final organ size are factors that control the timing of the cell proliferation arrest and the onset of cell expansion. The control of plant growth depends, in most cases, on an interplay between different hormones, which can affect either cell division (e.g. cytokinin), elongation (e.g. brassinosteroids, gibberellin) or, as auxin does, both processes [21]. Growth regulators, like the transcription factors *AINTEGUMENTA (ANT)* and *JAGGED*, the cytochrome P540 monooxygenase *KLUH* or the auxin-inducible gene *ARGOS* (through *ANT*) have a positive effect on petal cell proliferation, mainly via affecting its duration [22-25]. The restriction of the cell division period during petal development, on the other hand, is accomplished e.g. through protein degradation by the E3 ubiquitin ligase *BIG BROTHER* and by the predicted ubiquitin receptor *DA1* [26,27]. *MED25*, a component of the mediator complex, has a dual function in restricting the periods of cell proliferation as well as cell expansion [28]. Similarly, also *AUXIN-RESPONSE FACTOR8* is a negative regulator of cell division early in petal development and represses late cell expansion together with the transcription factor *BIG PETAL* [29,30]. In contrast, *ARGOS LIKE* promotes cell expansion [31].

Employing RNA-Seq, a *de novo* re-assembled petal transcriptome was generated for global analysis of *Iberis amara* petal development and to identify genes differentially expressed in the two petal pairs. This allowed detailed insight into the molecular network realizing unequal petal growth in the non-model organism *Iberis amara*. A significantly enhanced occurrence of genes involved in cell wall organization and modification processes in adaxial petals implies that the formation of smaller, adaxial petals is a result of an earlier onset of cell expansion, compared to the large, abaxial petals. In addition, microarray analyses of transgenic *Arabidopsis* plants ectopically expressing different *CYC2* transcription factors were performed in order to detect differences in target gene regulation.

## Results

### Analysis of spatial and temporal cell division patterns during *Iberis amara* petal development

**Figure 1:**
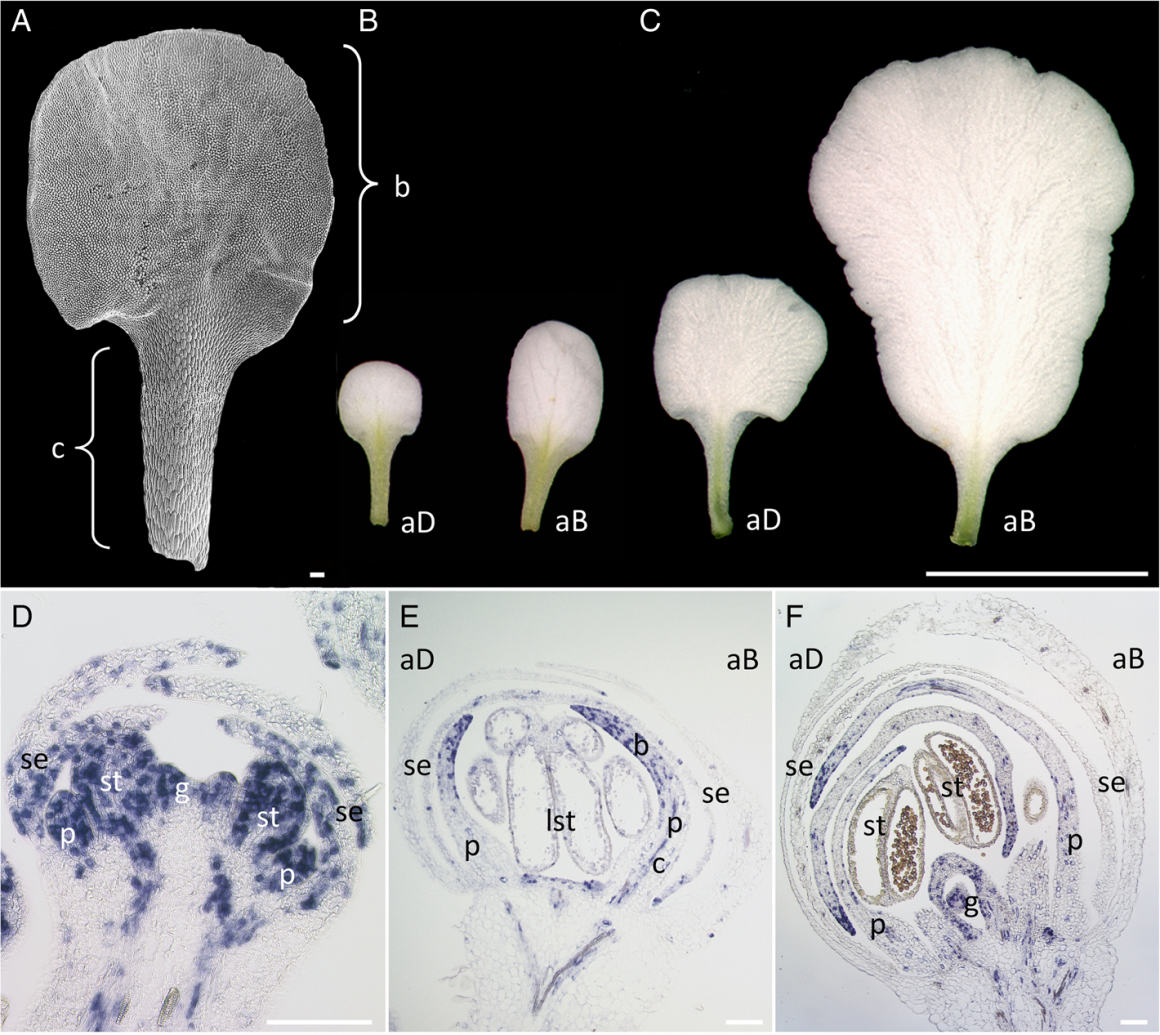
***Iberis amara*** **petal development. (A)** SEM image of adaxial stage A1 petal. Adaxial and abaxial stage A1 **(B)** and stage A2 **(C)** petals. **(D–F)**: *In situ* hybridization with *IaH4* of longitudinal sections through *Iberis amara* flower buds before anthesis. **(D)** In young flowers, *IaH4* is expressed throughout emerging petal buds. **(E)** Later, in differentiating petals, *IaH4* localizes to the upper, distal petal part that forms the blade and decreases in the proximal part, developing into the claw. **(F)** *IaH4* transcript accumulates before anthesis at petal tips in older flowers. A few cells scattered throughout the entire petal lengths still express *IaH4*. aD, adaxial; aB, abaxial; b, blade; c, claw; p, petal; se, sepal; lst, lateral stamen; st, stamen; g, gynoecium. Scale bars: **A, D-F**, 100 μm; **B** and **C**, 5 mm.

Both, adaxial and abaxial petals form an upper blade, with roundish conical epidermis cells and a lower claw, with elongated epidermal cells (Figure 1A). Elongated claw cells are greenish, indicating the presence of chloroplasts (Figure 1B and C). After the flowers open, unequal growth continues from stage A1 (Figure 1B) until the mature flower stage A2 (Figure 1C) and establishes the major size differences between the two petal pairs, likely via differential cell division, as described by [10].

The temporal and spatial cell division pattern during petal development was analysed by conducting *in situ* hybridisation studies in younger *Iberis* flower buds before anthesis with the cell cycle marker gene *Histone4* from *Iberis amara* (*IaH4)*. Initially, *IaH4* is expressed uniformly in petal primordia (Figure 1D). In older flowers, after the start of petal differentiation, *IaH4* expression declines in the proximal region and accumulates in the distal petal part (Figure 1E). This indicates that cell divisions cease first in the proximal region where cells expand into elongated claw cells, while cell divisions in the blade continue. Moreover, there are more cells expressing *IaH4* in the abaxial petal blade compared to adaxial petals (Figure 1E and F), as shown for an earlier stage by [10]. Around anthesis, when petals completely cover reproductive structures, *IaH4* expression concentrates predominantly at the distal petal tips and only few *IaH4* expressing cells are detectable in the proximal claw region (Figure 1F).

These data show that *Iberis* petals mature along a proximo-distal gradient with a decline in cell division moving from the base to the petal tip. Furthermore, cell division still persists around anthesis in the blade tips, promoting the establishment of the final 3.7-fold size difference between mature adaxial and abaxial petals.

### Reconstruction of the *Iberis amara* petal transcriptome

Prior to the detection of genes, which are differentially expressed in the *Iberis amara* corolla by RNA-Seq, a reference petal transcriptome was reconstructed. Two replicates were harvested from adaxial and abaxial petals of stage A1 flowers as *IaTCP1* shows at this stage the highest expression in adaxial petals and the largest expression difference between adaxial and abaxial petals [10]. In order to avoid genetic variance due to a lack of isogenic lines from this non-model organism that barely self-pollinates, all petal material was harvested successively from one individual plant.

The Illumina sequencing of all four libraries in total produced 184,515,960 quality filtered paired-end reads of approx. 50 nucleotides (nt) length, which resembles about 9 Gb of sequence data. Of those, 156,818,896 reads (about 85%) were used for the re-assembly (Table 1A), which is comparable to other RNA-Seq studies [32,33]. The 52,081 obtained contigs (Table 1B) could not be extended any more by concatamerization and, thus, represent the petal transcripts used for further analyses. The average contig size is 677 nt and ranges from 191 to 12,804 nt.

**Table 1:** ***De novo*** **assembly statistics**

**A**		
**Data**		**Read counts & nucleotides (Gb)**
Total reads (4 libraries)		184,515,960 (9.4)
Matched		156,818,896 (8.0)
Not matched		27,697,064 (1.4)
Percent assembled		85
**B**		
**Statistics of contigs**		
Contig number		52,081
Max sequence size (nt)		12,804
Min sequence size (nt)		191
Average sequence size (nt)		677
**C**		
**Contig length (nt)**	**Number of contigs**	**%**
100-499	30,739	59.02
500-999	11,326	21.75
1,000-1,999	7,435	14.28
2,000-4,999	2,457	4.72
5,000-9,999	120	0.23
> = 10,000	4	0.01
Total contig number	52,081	100.00

(A) Clean reads from all four libraries, pooled together, were used for the *de novo* re-assembly of the *Iberis* petal transcriptome. (B) Statistics of re-assembled contigs. (C) All contigs of the stage A1 *Iberis amara* petal transcriptome are classified into categories according to their nucleotide length. For each category, respective contig numbers and percentage of the total contig number are given. Gb, giga basepairs; nt, nucleotides.

About 59% of all contigs are smaller than 500 nucleotides, indicating that likely a larger portion of transcripts is not covered completely by their corresponding contigs. About 22% of all contigs have a size of up to 1,000 nt, 14% are between 1,000-2,000 nt long, followed by about 5% between 2,000-5,000 nt and 0.2% between 5,000-10,000 nt. Only four contigs are larger than 10,000 nucleotides (Table 1C). With the established petal transcriptome re-assembly a global analysis of petal development could next be accomplished.

### Annotation of *Iberis* genes involved in petal development

All 52,081 contigs were annotated based on BLASTX searches against an *Arabidopsis* protein database in order to allow a functional categorization of *Iberis* petal transcripts and to facilitate further comparison with *Arabidopsis* expression data. 45,001 *Iberis* contigs matched to a corresponding *Arabidopsis* Genome Initiative (AGI) code. 59.6% of matching contigs retrieved an AGI code that was found also as a match to other contigs. This is likely due to the fact that a large proportion of *Iberis* transcripts are not covered completely by their respective contigs and therefore several contigs were assigned to the same AGI code. Similarly, detection of an unexpected large number of genes encoding for transcription factors in *Marchantia* by RNA-Seq was explained as being caused by a “fragmentary” dataset, likely exceeding the number of actual transcription factors present [34]. For this reason, here, contigs with the same AGI code were treated as partial transcripts of the same gene and subtracted, yielding 18,139 putative unigenes. Next, applying an e-value cutoff of e^−4^ resulted in 15,983 (88%) likely unigenes with a putatively conserved function in *Arabidopsis*. Contigs without a hit might be either assembly artefacts or are unique to *Iberis*.

Based on their respective AGI codes, the 15,983 *Iberis* unigenes were grouped into functional categories provided by MapMan using the classification superviewer tool [35,36]. For an inter-species transcriptome comparison, microarray data from 13,492 genes expressed in *Arabidopsis* petals at stage 15 was taken. Stage 15 starts approximately one day after flower anthesis [37] and is, thus, closest in development to the *Iberis* stage A1, defined as the stage just after anthesis [10].

**Figure 2:**
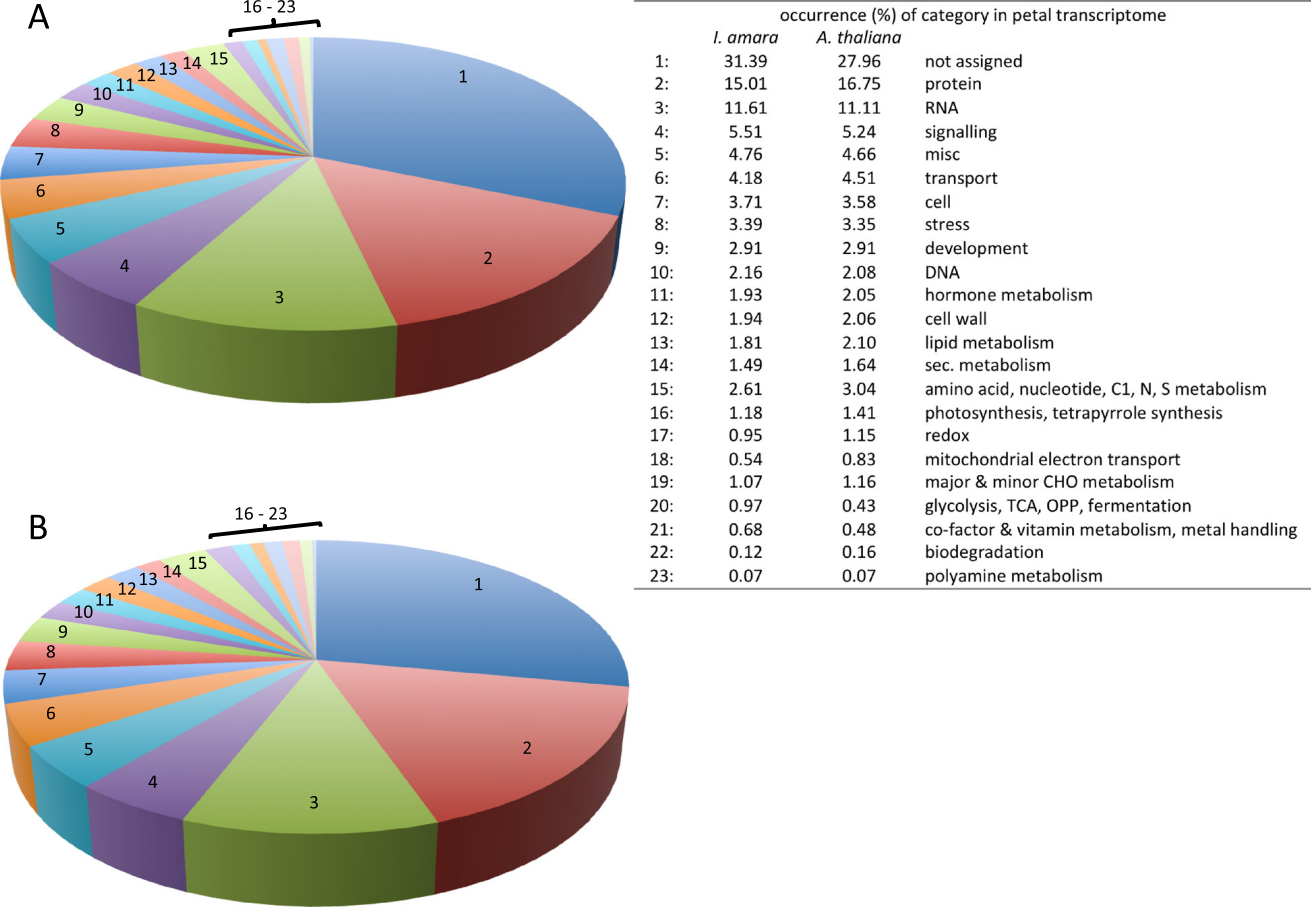
**Functional classification of the** ***Iberis amara*** **and** ***Arabidopsis thaliana*** **petal transcriptome.** Unigenes from *Iberis* and *Arabidopsis* petals were grouped into 23 functional categories. **(A)** Classification of 15,983 unigenes of the *Iberis amara* stage A1 petal transcriptome. **(B)** The *Arabidopsis* stage 15 petal transcriptome consists of 13,492 transcripts.

Both transcriptomes show an overall similar distribution of genes grouping into the respective categories, indicating that global crucifer petal development programs are similar (Figure 2A and B). Interestingly, the largest proportion of genes is classified as *not assigned*. This class constitutes 28% of the *Arabidopsis* petal transcriptome (Figure 2B) and 31% of all transcripts in the monosymmetric corolla of *Iberis* (Figure 2A). In addition, late petal development of both, *Iberis* and *Arabidopsis,* is governed predominantly by genes assigned to the categories *protein, RNA, signalling, misc* (subsuming various enzymes involved in different processes)*, transport* and *cell,* accounting together for about 75% of all categories.

### Characterization of *Iberis* petal development

In order to analyze gene expression levels in adaxial and abaxial petals, clean reads from the four samples were individually mapped to the *de novo* re-assembled petal transcriptome. Only reads that uniquely mapped to a single locus of the reference *Iberis* transcriptome were taken into account, amounting to 63–64% of all reads (Table 2). Gene expression levels are displayed as RPKM (reads per kilobase per million mapped reads) values for each sample library. After applying a cut-off p-value of 0.01 for multiplicative errors, 1,600 genes remained with reliable expression values. A complete list of all genes with their expression values is given in Additional file 1.

**Table 2:** **RNA-Seq reads and mapping statistics**

**Samples**	**aD_1**	**aD_2**	**aB_1**	**aB_2**
Total clean reads	25,793,295	42,131,488	28,273,758	38,725,897
Mapped reads	16,468,215	26,813,097	17,810,445	24,428,816
Mapped uniquely	**16,465,583**	**26,808,334**	**17,806,437**	**24,423,583**
Mapped non-specifically	2,632	4,763	4,008	5,233
Unmapped reads	9,325,080	15,318,391	10,463,313	14,297,081
Percent mapped uniquely	63.84	63.63	62.98	63.07

All clean reads obtained from the four libraries from adaxial (samples aD_1 and aD_2) and abaxial (samples aB_1 and aB_2) petals of stage A1 *Iberis* flowers were mapped against the re-assembled contigs. Only reads that map uniquely to one locus, highlighted in bold print, were used for the calculation of gene expression. This applies to 63–64% of total reads from the four libraries.

Amongst those 1,600 transcripts, the top 5% of strongest expressed genes from both petal types were functionally categorized to understand which biological processes are most profoundly required for late petal development in *Iberis* (Table 3, Additional file 2)*.* The category *misc* comprises the largest number of highly abundant transcripts. In addition, petal development seems to be dependent on the strong expression of genes that also exert roles in adaptation to biotic/abiotic stress and in hormone metabolism. Interestingly, transcripts of the aforementioned category *not assigned* are amongst the top 5% of strongest expressed genes in both adaxial and abaxial *Iberis* petals. This further corroborates the observation that late petal development seems to require the activity of a large number of thus far uncharacterized genes.

**Table 3:** **Functional categories of most abundant stage A1** ***Iberis*** **petal transcripts**

**Functional category**	**Number of contigs in**	
	**aD petals**	**aB petals**
Misc	15	13
Stress	9	9
Hormone metabolism	7	5
Not assigned	6	8

The 5% highly expressed genes from the 1,600 *Iberis amara* stage A1 adaxial (aD) and abaxial (aB) petals unigenes were assigned to their functional MapMan categories. Categories present in both samples are listed, together with the number of genes from each sample that belong to the respective category.

### Identification of transcripts differentially expressed in adaxial and abaxial petals

For all 1,600 genes, expression values in adaxial and abaxial petals were compared against each other (Table 4A), resulting in 1,141 genes being stronger expressed in adaxial petals and 459 transcripts with higher RPKM values in abaxial petals. The majority, 1,266 genes, have an expression fold change less than 2-fold, with 828 being higher expressed in adaxial petals and 438 in abaxial petals. The expression of 334 genes is changed two-fold or more between the two petal types, with 313 genes being more abundantly transcribed in adaxial petals and only 21 genes being more active in abaxial petals. Most of these genes are differentially expressed up to a fold change of five (240 in adaxial and 20 in abaxial petals). Only a small fraction displays a fold change equal to or above five (64 and 9 in adaxial petals and one gene in abaxial petals), including also *IaTCP1*, which is about 27-times upregulated in adaxial petals, confirming previous observations [10] (Additional file 3).

**Table 4:** **Classification and analysis of genes differentially expressed between adaxial and abaxial petals**

**A**					
**Fold change**	**# Genes**	**↑ Adaxial petals**	**↑ Abaxial petals**		
1 - 1.9	1,266	828	438		
2 - 4.9	260	240	20		
5 - 9.9	65	64	1		
> 9.9	9	9	0		
Total numbers	1,600	1,141	459		
Fold change ≥ 2	334	**313**	21		
**B**					
**Functional category (contig nr)**					
Not assigned (81)					
Misc (26)					
Protein (25)					
Transport (24)					
**Signalling (23)**					
RNA (23)					
**Cell wall (21)**					
**Photosynthesis (16)**					
Stress (16)					
**C**					
**GO term**	**aD petal genes**	**%**	**Petal genes**	**%**	**Fold enrichment**
Cell wall organization	15	4.8	180	1.1	4.2
Cell wall modification	9	2.9	73	0.5	6.3
Cell-cell signalling	5	1.6	17	0.1	14.6
Flower morphogenesis	3	1.0	5	0.0	32.0
Endocytosis	5	1.6	27	0.2	9.4
Membrane budding	5	1.6	16	0.1	16.0
Clathrin coat assembly	5	1.6	13	0.1	20.0
Photosynthesis (phs)	17	5.4	148	0.9	5.8
phs, light reaction	10	3.2	71	0.4	7.3
phs, light harvesting	6	1.9	22	0.1	13.7
Carbon fixation	4	1.3	17	0.1	11.6

(A) All 1,600 unigenes were classified according to the strength of their expression fold change between adaxial and abaxial petals. (B) 313 unigenes, upregulated in adaxial petals (≥2-fold), were assigned to their respective functional MapMan catagories. Numbers of genes assigned to respective categories are bracketed. Categories that appear also in (C) are highlighted in bold print. (C) Significantly enriched functional categories amongst 313 unigenes, which are upregulated in adaxial petals in comparison to the general *Iberis* petal transcriptome were determined using FatiGO [38]. For each GO term, the number of genes assigned to the respective GO term amongst the 313 upregulated adaxial petal genes and the 15,983 unigenes forming the *Iberis* petal transcriptome, are given. Numbers are also expressed in percent, together with the respective fold enrichment.

Overall, the data indicate that a significantly higher gene number governs the development of small adaxial petals compared to the control of large abaxial petal formation.

### Determination of gene categories in the adaxial petal transcriptome

To get an overview of developmental processes participating in the formation of the two petal types, differentially expressed genes with a minimal fold-change of two were classified into functional MapMan categories. No categories that consisted of more than three members, one of the applied threshold criteria, were found for the 21 transcripts upregulated in abaxial petals. Amongst the 313 transcripts upregulated in adaxial petals, about 25% (81) were not assigned into any category, encoding for proteins with unknown functions (Table 4B and Additional file 3). A large number of genes (26) is grouped together as encoding for various enzymes in the category *misc* and 25 transcripts are involved in *protein* metabolism, such as protein modification, targeting and degradation. The category *transport* comprises 24 genes, encoding for proteins transporting inorganic compounds, primary metabolites, ions, metal or auxin. Categories *cell wall, signalling* and *photosynthesis* were also detected as being significantly enriched when applying gene ontology (GO) terms of AGI codes linked to the adaxial-specific transcripts and are described below. Amongst the category *RNA* (23 members), 20 encode for various transcription factors, including *IaTCP1*. In addition, 16 adaxial upregulated genes perform a role in the adaptation to biotic and abiotic stress.

To gain a more detailed understanding of the developmental mechanisms and the respective genes involved in generating smaller adaxial petals, significantly enriched GO terms amongst the 313 adaxial-specific transcripts were revealed using FatiGO [38].

Individual genes are repeatedly allocated to related GO terms. GO terms associated to cell wall-related processes, such as *cell wall organization* and *modification* are significantly enriched in adaxial petals (Table 4C). The in total 15 genes exerting cell wall-related functions encode for pectate lyases, pectin methyl esterases/inhibitors implicated in pectin degradation [39] and other enzymes that function in cell wall remodelling and loosening. Even more genes were assigned to the MapMan category *cell wall* (21) (Table 4B) and include, in addition to cell wall degrading/modifying enzymes, COBRA like 10 and an arabinogalactan protein. Both proteins constitute a putative link between the cell wall and the cytoplasm [40].

The GO term *cell-cell signalling* contains five transcripts encoding for small signal peptides that play a role in plant cell growth (Table 4C). These peptides were first discovered in tobacco leaf extracts due to their ability to induce a rapid pH increase in cell suspension cultures and were, hence, termed RALFs (rapid alkalinization factors) [41,42].

The GO term *flower morphogenesis* includes three transcription factors involved in petal development, namely *PETAL LOSS*, *BLADE ON PETIOLE 1* and *2*. The categories *endocytosis, membrane budding* and *clathrin coat assembly* include genes that encode for ENTH/ANTH/VHS superfamily proteins, key regulators of the endosomal system involved in membrane trafficking [43].

Unexpectedly, 17 genes were found to be associated with photosynthesis, including the GO terms *photosynthesis, light harvesting*, *light reaction* and *carbon fixation.* In order to analyse whether photosynthesis-related processes contribute to flower monosymmetry formation in *Iberis*, this finding was scrutinized by detailed qPCR analyses. Therefore, a phosphoribulokinase homolog from *Iberis* was analysed as a representative for the category *photosynthesis* and confirmed the RNA-Seq data showing a stronger expression in adaxial petals (Additional file 4A). Next, qPCR was performed on separately harvested petal blade and claw material of the two petal types to test if the greenish claw cells, which are present in both petal types (Figure 1B), contribute to the detection of photosynthesis-related genes. The phosphoribulokinase transcript is not differentially expressed between adaxial and abaxial petal blades or claws (Additional file 4B), implying that photosynthesis genes do not contribute to the formation of smaller adaxial petals. In both petal types, the greenish claw region, where the phosphoribulokinase is stronger expressed (Additional file 4C), is similar in size and shape but blade sizes differ significantly. Therefore, the differential expression of photosynthesis-related genes detected with RNA-Seq might be due to the fact that harvesting whole petals diluted out claw-expressed genes stronger in large abaxial petals compared to small adaxial petals. As a consequence, photosynthesis-related genes were excluded from further analyses.

### Expression analysis of genes upregulated in adaxial *Iberis* petals via qPCR

**Figure 3:**
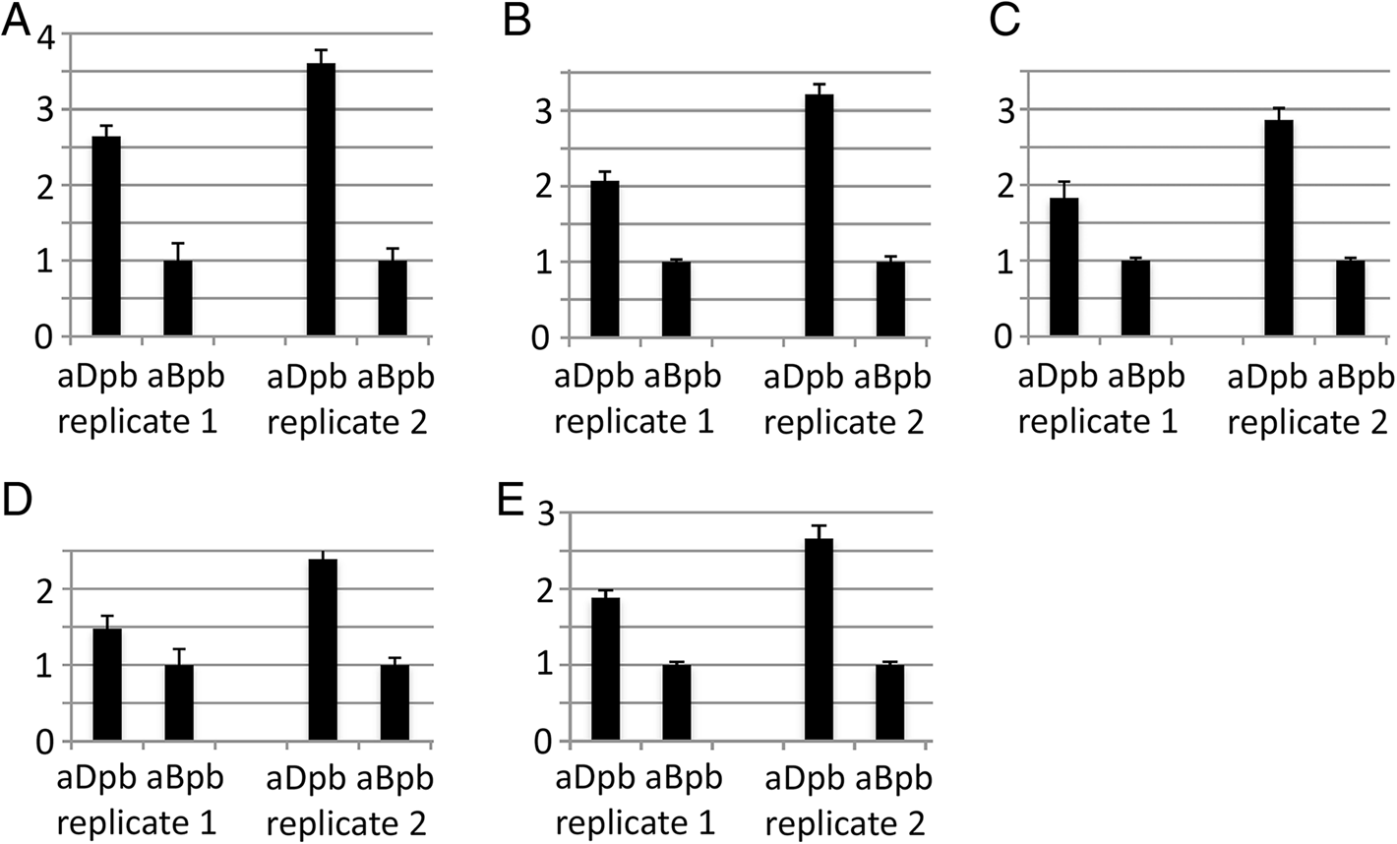
**Differential expression of cell wall-related genes in adaxial versus abaxial petal blades.** Five genes involved in *cell wall modification/organization* were chosen as representatives for further qPCR analysis: **(A)** a *VANGUARD1* homolog 2, **(B)** a polygalacturonase, **(C)** a glycosyl hydrolase, **(D)** a pectate lyase and **(E)** an invertase/pectin methylesterase inhibitor. Stage A1 adaxial and abaxial petals from two biological replicates (replicate 1, replicate 2) were dissected into upper blade and lower claw and expression of the respective transcripts was compared only between adaxial and abaxial petal blades. aBpb, abaxial petal blade; aDpb, adaxial petal blade.

**Figure 4:**
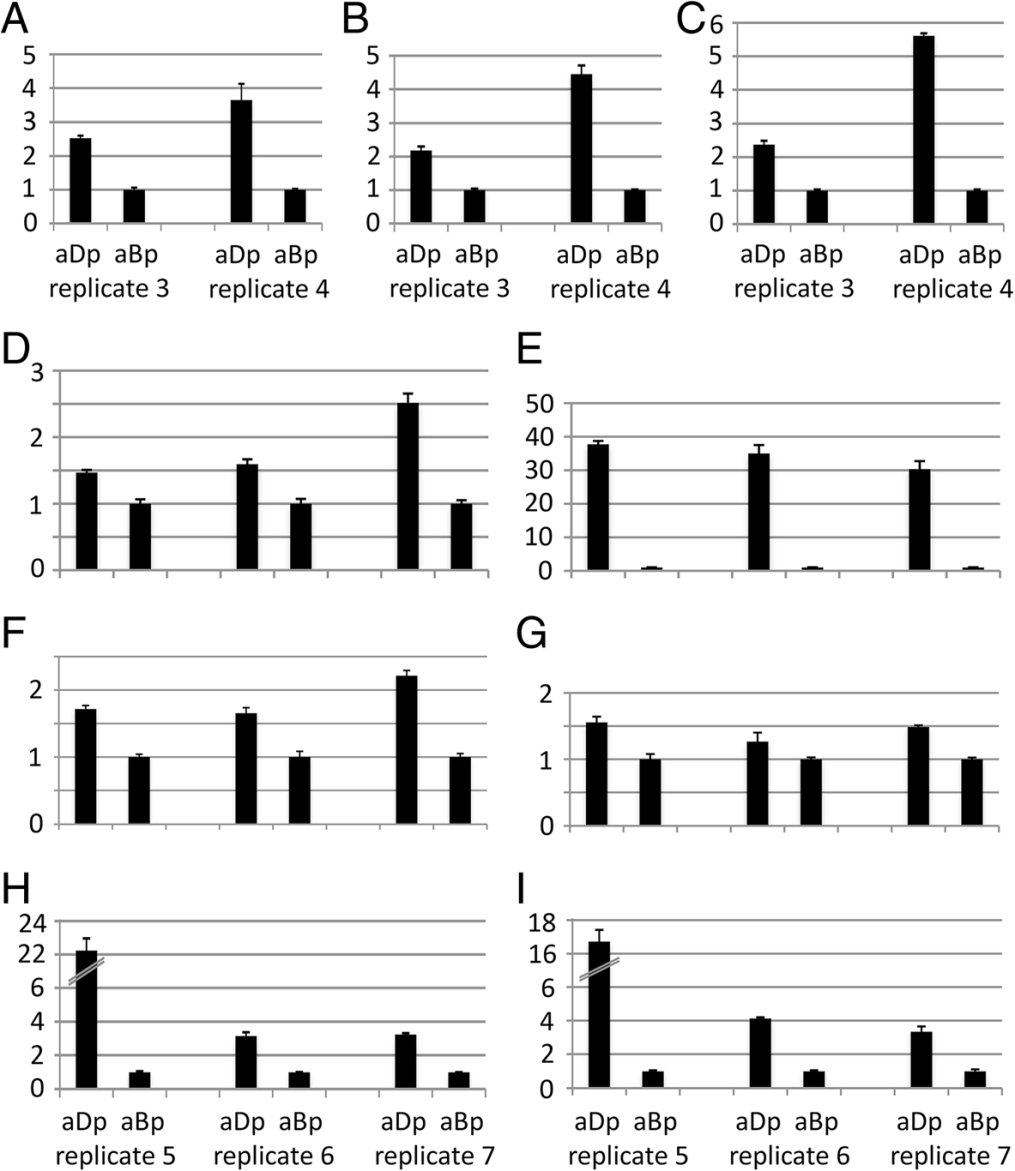
**qPCR of selected genes with a stronger expression in adaxial petals.** Expression analysis via qPCR was carried out on adaxial and abaxial petals from two (**A**-**C**, replicate 3, replicate 4) and three (**D**–**I**, replicates 5–7) different biological replicates. **(A)** *RALF-like 15*, **(B)** *RALF-like 19*, **(C)** *COB-like 10*, **(D)** *PETAL LOSS*, **(E)** *IaTCP1*, **(F)** *P-glycoprotein 13*, **(G)** *cytokinin oxidase 3*, **(H)** *Rcd1-like* and **(I)** a contig with homology to a protein of unknown function (At5g42680). aBp, abaxial petal; aDp, adaxial petal.

Next, transcripts from other significantly enriched GO terms (Table 4C) were further analysed by qPCR. From the category *cell wall organization* five representatives with a likely function in cell size control were selected. All cell wall-related genes were tested exclusively on blade material at stage A1 of the two petal types, to exclude effects mediated by elongated claw cells (Figure 1A). All tested *Iberis* genes, a *VANGUARD1* homolog 2, a polygalacturonase, a glycosyl hydrolase, a pectate lyase family member and an invertase/pectin methylesterase inhibitor show a 1.5 to 3.6-fold stronger expression in adaxial petal blades compared to abaxial ones (Figure 3A-E), implying an enhanced participation of cell wall related processes in the formation of smaller adaxial petals. From the category *cell-cell signalling* (Table 4C) two out of the five transcripts were selected for further qPCR analysis. Both *Iberis RALF* genes are stronger expressed in adaxial petals (Figure 4A and B), indicating a possible function of this group of *cell-cell signalling* genes in the formation of small petal size. The trihelix transcription factor *PETAL LOSS* (*PTL*) belongs to the significantly enriched category *flower morphology* (Table 4C) and shows an enhanced expression in adaxial petals (Figure 4D). Also, the strong differential petal expression of the *TCP* transcription factor *IaTCP1* [10] was verified by qPCR and shows the strongest expression difference between the two petal types amongst all genes tested (Figure 4E).

From these data, a general picture emerges, where adaxial petals are characterized by a pool of transcripts enriched in genes involved in cell wall organization and signalling. This implies that smaller petals in comparison to larger petals are the result of an earlier stop of cell division. Concomitantly, up-regulation of genes participating in cell elongation and differentiation occurs earlier in the small, adaxial petals compared to the larger, abaxial petals, the latter remaining longer in the cell proliferation phase. Further genes were selected from transcripts upregulated in adaxial petals (Additional file 3) based on their annotation and a putative relation to such processes.

A first interesting candidate for a participation in cell expansion processes is *COBRA-like 10 (COBL10)*, a member of the *COB* multigene family. *COBRA*, the first analysed member, is strongly expressed in the root elongation zone and was proposed to be a key regulator of oriented cell expansion [44]. Similarly, *COBL10* is involved in the control of the directional and likely female-guided pollen tube growth [45]. In both biological samples, the *Iberis COBL10* is upregulated in adaxial petals compared to abaxial petals (Figure 4C), as shown with RNA-Seq. Another gene upregulated in adaxial *Iberis* petals is *P-glycoprotein (PGP) 13* (Figure 4F), which belongs to the plant ABC transporter superfamily from the category *transport* (Table 4B). *PGP*s have been shown to fulfil a role in plant growth and development as they mediate the cellular and long-distance transport of auxin [46]. *Cytokinin oxidase 3* (*CKX 3*) catalyzes the breakdown of the plant hormone cytokinin and by that exerts a negative influence on cell division [47]. Therefore, *CKX 3* with its RNA-Seq expression fold change of 1.9 being slightly below the two-fold threshold was included into further qPCR analysis. The stronger expression of the *Iberis CKX 3* homolog in adaxial petals (Figure 4G) might be involved in reducing the rate of cell division, prior to the switch into cell elongation. Also *Rcd1-like*, a protein involved in cell differentiation from the category *RNA* (Table 4B) shows a higher expression in adaxial petals (Figure 4H). The mammalian *Rcd1* gene is a transcriptional cofactor with a role in the transition of cells to differentiate [48]. The stronger expression in adaxial petals of a gene that shows highest homology to the *Arabidopsis* gene At5G42680 encoding for a protein of unknown function (Figure 4I), confirms the differential expression of a member of this highly abundant class of transcripts upregulated in adaxial petals.

In summary, qPCR analysis strengthens the observation that differential petal size development is accomplished via an earlier onset of cell elongation and differentiation in smaller adaxial petals, as indicated by an upregulation of genes that are involved in cell wall remodelling, repression of cell division, cell differentiation and signalling.

### Interspecies comparison of *CYC2* target genes via ectopic *CYC2* expression in the heterologous system *Arabidopsis thaliana*

Transgenic *Arabidopsis* plants overexpressing the two crucifer *CYC2* transcription factors *IaTCP1* and *TCP1* produce flowers with smaller petals, whereas ectopic expression of *CYC* from *Antirrhinum* results in the formation of larger petals [10,13]. To compare the effect of the CYC, IaTCP1 and TCP1 proteins on downstream regulatory networks in *Arabidopsis*, a microarray analysis was conducted. Material for RNA extraction was harvested from young inflorescences, comprising flower bud stages before anthesis, of transgenic T2 plants overexpressing these genes.

**Figure 5:**
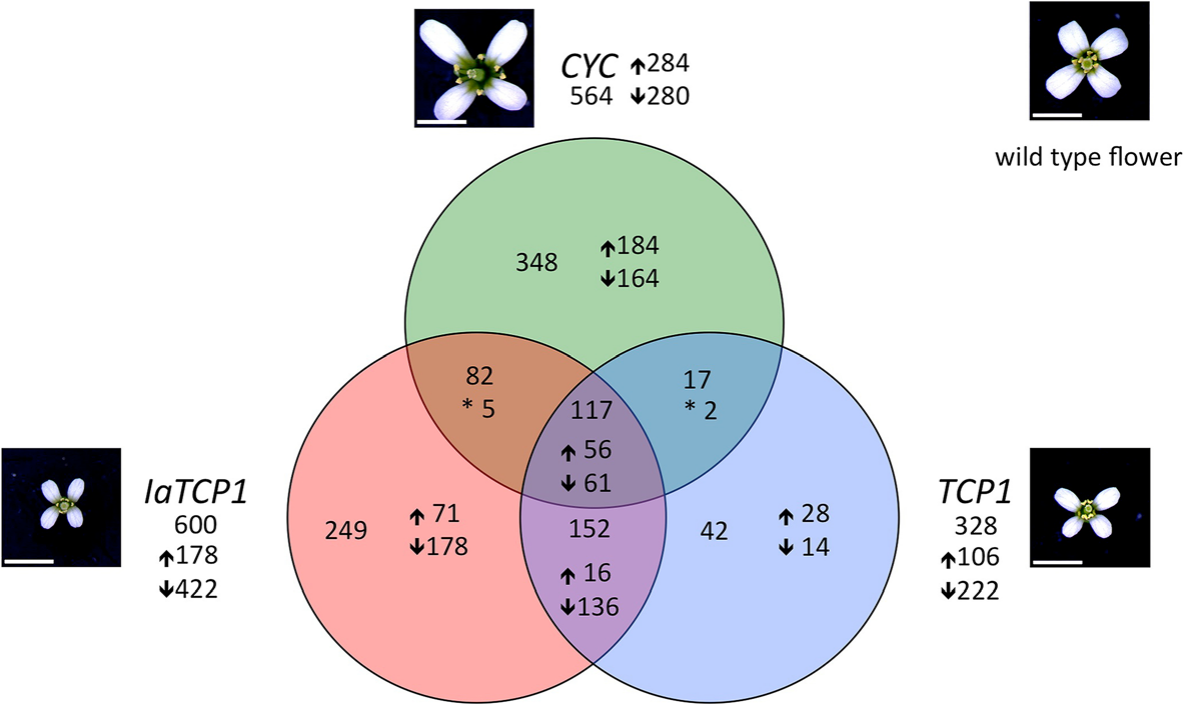
**Numbers of differentially expressed genes caused by ectopic activity of** ***CYC2*** **transcription factors.** The diagram shows the numbers of differentially expressed genes detected with microarrays hybridized with inflorescence cDNA from transgenic *Arabidopsis* plants overexpressing either *IaTCP1* (red), *CYC* (green) or *TCP1* (blue), together with respective floral phenotypes. Total target gene numbers are given outside the circles, numbers of target genes solely addressed by respective transcription factors are shown inside the circles. Numbers of genes that are mutually addressed by two or all three *CYC2 t*ranscription factors are displayed in intersections. Arrows indicate up- or downregulation, asterisks denote mutually addressed genes with a reciprocal expression. Scale bars: 2 mm.

In total, 600 genes were determined to be differentially regulated following ectopic *IaTCP1* expression, the majority (422) being negatively affected (Figure 5). Slightly less genes (564) respond to an overexpression of *CYC* (284 up- and 280 down-regulated). The lowest number of genes (328) is addressed by ectopic *TCP1* activity (106 up- and 222 downregulated).

The *CYC2* transcription factors *CYC* and *IaTCP1*, which govern monosymmetry development, address both a larger number of target genes in the heterologous *Arabidopsis* system than *TCP1*. This tendency is more obvious regarding only the number of target genes, which are exclusively addressed by a single CYC2 protein, namely 348 by CYC, 249 by IaTCP1 and only 42 by TCP1. The capability of the *CYC* and *IaTCP1* genes to address a larger number of downstream targets might reflect their function in the control of monosymmetry formation, even when expressed in a heterologous species forming a polysymmetric corolla such as *Arabidopsis*. 117 target genes are commonly addressed by all three transcription factors and might thus not be linked to specific functions in symmetry regulation. The highest number of overlappingly regulated genes is found between the two crucifer transcription factors (152), whereas the smallest number of targets is mutually addressed by CYC and TCP1 (19).

To gain insight into the molecular network acting downstream of *IaTCP1*, significantly accumulated GO terms were detected. For the 422 genes repressed by *IaTCP1*, the majority of enriched GO terms are associated with cell wall-related processes (*cell wall organization, cell wall modification, plant type cell wall organization*, *unidimensional cell growth* and *plant type cell wall loosening*). Other enriched GO terms are associated with *secondary metabolic processes*, *H*_*2*_*O*_*2*_*catabolic processes* and *response to red light* (Table 5). Additional file 5 summarizes single genes, amongst them 11 cell wall genes that are mutually addressed by ectopic expression of *IaTCP1* in *Arabidopsis* inflorescences and by endogenous *IaTCP1* in adaxial *Iberis* petals.

**Table 5:** **Enriched GO terms in the pool of** ***IaTCP1*** **target genes in** ***Arabidopsis*** **inflorescences**

**GO term**	**Genes ↓ by IaTCP1**	**%**	**Arabidopsis infl. genes**	**%**	**Fold enrichment**
Cell wall organization	21	5.0	141	1	5.0
Cell wall modification	11	2.6	55	0.4	6.7
Plant type cell wall org.	7	1.7	34	0.2	6.9
Unidimensional cell growth	11	2.6	90	0.6	4.1
Plant type cell wall loosening	5	1.2	16	0.1	10.7
sec. metabolic process	21	5.0	233	1.7	3.0
H_2_O_2_ catabolic process	6	1.4	27	0.2	7.5
Response to red light	7	1.7	36	0.3	6.4

Listed are GO terms that are significantly enriched amongst all genes negatively regulated upon *IaTCP1* overexpression in *Arabidopsis* inflorescences in comparison to the *Arabidopsis* inflorescence transcriptome. For each GO term, numbers of genes clustering to these categories from the pools of 422 downregulated genes and from the 14,064 genes forming the *Arabidopsis* inflorescence transcriptome, are given. Numbers are also expressed in percent, together with their respective fold enrichment.

RNA-Seq and microarray analyses, both, detected a differential regulation of genes involved in cell wall processes. However, in young transgenic *Arabidopsis* inflorescences all cell wall-related transcripts are downregulated following an ectopic *IaTCP1* activity, whereas they are upregulated in older *Iberis* petals after anthesis, in correlation with and presumably also in response to a high endogenous *IaTCP1*-expression. This indicates that the spatial and temporal context of *CYC2* activity is crucial for its proper effect on cell growth and differentiation.

## Discussion

### Monosymmetry in *Iberis* is achieved through differential cell proliferation during late flower development

*Iberis* petals are initiated simultaneously and unequal growth begins around the onset of stamen differentiation, from where it proceeds and gains its maximal size difference after anthesis, likely driven by differential cell proliferation [10]. This requires cell division to continue throughout later phases of flower development, which was demonstrated by monitoring the cell division pattern via *Histone4* expression from *Iberis* in different petal stages. In older petals, cell division still takes place close to anthesis in both petal types, where it is restricted mainly to the petal tips. Similar to *Iberis*, cell division in *Arabidopsis* petals increases throughout flower development until stage 11 and can still be visualized in stage 12, just before anthesis [23].

After anthesis, adaxial *Iberis* petals increase 2.8-fold, whereas abaxial petals increase 6.7-fold to reach their final size. In contrast, cells of both petal types only double in size from anthesis to maturity [10]. These data indicate that cell division – at least in abaxial petals – proceeds beyond anthesis.

### Transcriptome analysis of petal development of *Iberis amara*

RNA-Seq is being used more and more often as a convenient tool for comparative transcriptome analyses of species with inavailable genome sequences [49-51]. Applying an RNA-Seq approach, we investigated the *Iberis* transcriptome at the late petal developmental stage A1, just after anthesis of flowers.

Comparison of the *Arabidopsis* and *Iberis* petal transcript distribution into functional categories indicates that molecular processes governing late petal development are similar in the two crucifers. Interestingly, for the largest group of the *Iberis* petal transcriptome, comprising 31% of the transcripts, no function could be assigned yet. A similar situation with 28% of these genes is observed for *Arabidopsis*. Interestingly, comparative analysis of different petal stages in *Antirrhinum*, where flowers form an even more elaborate and complex shaped corolla, revealed that the frequency of the unclassified protein group accounts for over 50% amongst all transcripts expressed in petals [16]. This suggests that a large proportion of genes exert an intriguing, yet uncharacterized, function during petal morphogenesis.

Analysis of the most abundant transcripts in *Iberis* petal stage A1 revealed that various enzymes, subsumed in the category *misc*, are highly expressed in the two petal types, amongst them GDSL-lipases and lipid transfer proteins. Both enzyme types have a function in lipid metabolism and have been described as putative target genes of the petal and stamen-specifying B-class MADS box transcription factor during petal development in *Antirrhinum* and *Arabidopsis* [16,52]. The cytochrome P450 gene *CYP76C3*, which is amongst the most highly expressed *Iberis* petal genes, is also strongly expressed in various *Arabidopsis* floral organs, where it participates in the linalool metabolism. Linalool, a well-studied attractant for pollinators, is converted further into its oxigenated derivates by CYP76C3 in autogamous *Arabidopsis* flowers [53], which likely function in plant defence against pathogens and insect herbivores [54]. Two lipoxygenases, *LOX1* and *LOX2*, which exert functions in hormone metabolism, are also highly expressed in *Iberis* petals. The LOX2 protein catalyzes the first dedicated step in jasmonic acid (JA) biosynthesis and is activated by the transcription factor TCP4 in leaves [55]. *TCP4* is also expressed in both *Iberis* petal types (Additional file 1), and since LOX2 is localized to chloroplasts [56], *LOX2*-dependent induction of JA likely takes place in the green petal claws, where JA might exert a negative effect on cell proliferation [57].

### The monosymmetric corolla formation of *Iberis* is realized through differential timing of the switch from cell division to cell elongation

With almost 15-times more genes upregulated in the small adaxial petals compared to the large abaxial petals, it seems that more genes are required to restrict adaxial petal size during *Iberis* corolla development. The fact that the monosymmetry key regulator *IaTCP1* is highly expressed in adaxial petals and represents one of the genes with the largest expression difference implies that IaTCP1 activates a larger number of target genes compared to its inhibitory transcription effects.

Differential *Iberis* petal development is likely driven by unequal cell division processes. Consistently, genes exerting negative effects on cell division are amongst the pool of transcripts upregulated in adaxial petals. In *Arabidopsis, cytokinin oxidase (CKX) 3* catalyses the metabolic breakdown of the plant hormone cytokinin and plants with defective *ckx* genes form flowers with enlarged petals due to an increased cell number [47]. The trihelix transcription factor *PTL* is expressed in regions of developing *Arabidopsis* flowers, where growth is repressed. *PTL* expression in the inter-sepal zone is assumed to keep cell proliferation under control to allow the outgrowth of petal primordia. The loss of *PTL* function results in an increased cell proliferation in that region [58]. Petals are initiated in response to localized auxin accumulation, and Lampugnani and colleagues [59] speculate this could be evoked via a supportive role of *PTL* in non-polar auxin efflux through PGPs. PGPs belong to the superfamily of ABC-transporters and function in mediating auxin transport [60]. *PGP13*, which is also transcriptionally enriched in adaxial *Iberis* petals, belongs to the same clade as the auxin exporters *PGP1* and *PGP19* [46], for which double mutant plants show severe growth defects [60].

In addition to transcripts negatively affecting cell proliferation, a large number of genes related to cell wall modifying processes are upregulated in adaxial petals. After an initial phase of organ growth by cell division, final organ size is realised by cell elongation [14,15]. The primary cell wall of land plants is composed of a strong cellulose microfibril framework cross-linked through xyloglucan (XG) chains and embedded in a stabilizing matrix of pectin polysaccharides and structural proteins. Plant cell walls provide stability by withstanding tensile stress, and cell growth results from changes in wall extensibility by relenting to this stress [61,62]. Amongst the group of cell wall modifying proteins in adaxial *Iberis* petals are several pectinases, enzymes that function in the de-/methylesterification or degradation of pectin [39] and, by that, might contribute to cell growth via precisely tuning the proportion of pectate in the cell wall [62]. A *Xyloglucan Endotransglucosylase (XET)*, which cleaves and re-attaches xyloglucan chains *in vitro* was found to be upregulated in adaxial petals. *XET* activity correlates with regions undergoing cell expansion, indicating that *XETs* have a role in cell expansion, e.g. through the biogenesis of newly formed cell walls (reviewed in [63]). In addition, several *Iberis glycosyl hydrolases (GHs)* were upregulated in adaxial petals, which form a diverse group of enzymes that hydrolyze glycosidic bonds [64]. One *Arabidopsis* homolog of the *GHs* belongs to the subfamily of the endo-1,4-ß-glucanases *KORRIGAN*, which is essential for cell elongation and cellulose synthesis in *Arabidopsis* hypocotyls [65]. Several transcripts encoding for a COBRA-like protein, two proline rich extension-like receptor kinases (PERKs), an arabinogalactan protein and a formin are upregulated in adaxial petals and might act as a potential connection between the cytoplasma and cell wall. These proteins are, thus, part of the molecular continuum that connects the cytoskeletal network, the cytoplasm and the cell wall, and which contributes to cell expansion control in *Arabidopsis* (reviewed in [40]).

*Arabidopsis* homologs of several of the floral cell wall-related genes, such as a polygalacturonase (At1G02790), a XET (At1G65310) and a pectin lyase like member (At5G07430) are also predominately expressed in the cell elongation zone of root tips (visualized with the *Arabidopsis* eFP browser at bar.utoronto.ca) and only weakly in the preceeding meristematic region, where growth is realized by cell divisions. This supports their general function in cell wall modification, both in a floral and root context, contributing to tissue growth by mediating cell elongation processes.

The determination of final organ size requires the precise coordination of both, cell division and cell elongation processes, and likely depends on a tight cellular communication. Amongst all adaxial-petal specific genes, a group of transcripts involved in cell-cell signalling is significantly enriched. These RALFs are small peptides, which are important for many developmental processes and regarded as novel plant hormones [41]. In grass leaves, RALFs were found to be mainly expressed in young elongating cells beyond the meristematic zone, and expression is almost absent in mature cells [66]. As RALFs are thought to exert a negative effect on cell growth, their expression strength and location is likely precisely adjusted during organ development to achieve an accurate guidance of cell expansion without completely blocking it [66,67].

Summing up, a significantly increased number of transcripts involved in cell wall modification and cell-cell signalling, together with genes regulating the restriction of cell division and auxin transport were detected in the pool of 313 transcripts enriched in small, adaxial *Iberis* petals. Hence, unequal petal growth is likely achieved via an earlier stop of cell division in adaxial petals with a subsequent earlier onset of cell expansion, whilst abaxial petal cells continue dividing. In *Iberis*, cell division ceases in an acropetal gradient, where cells at the tip keep on dividing longest, similar as in other plant species [23,68]. Thus, an unequal stop of cell division might be realised through a faster acropetal progression of a cell division stop front in adaxial petals compared to abaxial petals.

### Ectopic expression of *IaTCP1* causes expression changes of cell wall related genes in *Arabidopsis*

Microarray analyses aimed at comparing the molecular networks that are addressed in inflorescences by *IaTCP1* and *TCP1* causing smaller petal formation, and by *CYC*, giving rise to the development of larger petals.

A considerable number of genes (117 out of 600, 564 and 328, respectively) is addressed by all three CYC2 proteins together. These might be transcripts that constitute a common basal set of *TCP/CYC2* transcription factor targets genes in *Arabidopsis thaliana*. Interestingly, in the heterologous *Arabidopsis* system, more genes are exclusively addressed by *IaTCP1* from *Iberis* (249) and *CYC* from *Antirrhinum* (348), developing a monosymmetric flower, than by *TCP1* from *Arabidopsis* (42), forming a polysymmetric corolla. These quantitative differences indicate that *IaTCP1* and *CYC* might regulate more target genes, implying that a larger downstream regulatory network realizes monosymmetric compared to polysymmetric corolla development. Therefore, in addition to cis-regulatory changes that led to a heterochronic *CYC2* gene expression shift in the Brassicaceae, promoting corolla monosymmetry evolution [10,12], also a divergence of trans-acting *CYC2* proteins likely contributed to the establishment of this morphological novelty.

The largest overlap in target gene number is shared between *IaTCP1* and *TCP1*. These 152 genes comprise several transcripts involved in cell wall-related and signalling processes (Additional files 6 and 7) and the activity of these exclusive crucifer targets could be essential for the petal size restriction in *Arabidopsis*, as only transgenic plants ectopically expressing a crucifer CYC2 protein develop flowers with smaller petals [10]. Amongst the population of target genes downregulated in response to heterologous *IaTCP1* expression in *Arabidopsis*, again a high proportion is involved in cell wall modification processes. This indicates that *IaTCP1* addresses these genes in *Arabidopsis* and that the high abundance of this gene class detected with RNA-Seq in adaxial *Iberis* petals is the effect of the strong *IaTCP1* expression there. While cell wall genes are positively affected in older adaxial *Iberis* petals, where *IaTCP1* expression is strongly enhanced, *IaTCP1* activity has a negative effect on cell wall genes in young *Arabidopsis* inflorescences overexpressing *IaTCP1*. TCP transcription factors are known to exert either an activating or repressive effect on organ growth, depending on their tissue specific expression [7,13,69]. As TCP proteins act as dimers [70], the outcome of their activity might differ in dependence of available interaction partners in different organs or developmental stages. Similar development-dependent regulatory effects were shown previously for the KNOX/BEL transcription factor BELLRINGER [71] (BLR), which interacts with other BEL and KNOX proteins [72,73]. One of its targets, the pectin methylesterase *PME5* is repressed by BLR in the shoot meristem, to allow coordinated formation of primordia, but is activated by BLR in internodes, where it promotes cell elongation [71].

## Conclusions

An RNA-Seq expression analysis on adaxial and abaxial stage A1 *Iberis* petals reveals a significantly increased number of cell wall-related transcripts in adaxial petals. This indicates that differential petal growth, a characteristic of the monosymmetric *Iberis* corolla, is achieved through an earlier stop of the cell proliferation phase followed by the onset of cell expansion and differentiation in the smaller adaxial petals, while abaxial petal cells likely still continue growth via cell division. As ectopic expression of *IaTCP1* in *Arabidopsis* addresses cell wall-related genes, the enhanced occurrence of this gene class in adaxial *Iberis* petals is very likely also a direct consequence of the strong *IaTCP1* expression there. Moreover, crucifer monosymmetry evolution likely relied on the combination of cis-regulatory changes, leading to a late adaxial *IaTCP1* expression and also on trans-regulatory changes mediated by differences in the CYC2 proteins themselves, altering the set of potential target genes that contribute to the large petal size differences in *Iberis amara*.

## Methods

### Plant material and growth conditions

*Iberis amara* seeds were purchased from Saatgut Kiepenkerl (Everswinkel, Germany) and have been cultivated in the greenhouses for several generations. Plants were grown under standard greenhouse conditions at the University of Osnabrück. Transgenic *Arabidopsis thaliana* (Col.) plants were grown at 20°C with 13-hrs light/11-hrs dark cycles in growth chambers.

### Illumina sequencing, *de novo* assembly and RNA-Seq analysis

For RNA-Seq, two technical replicates of stage A1 adaxial (samples aD_1 & aD_2, Table 2) and abaxial (samples aB_1 & aB_2, Table 2) petals were harvested from one single plant, in order to avoid genetic variation, that would hinder the subsequent transcriptome *de novo* assembly. Plant material was flash frozen in liquid nitrogen and total RNA was isolated with the RNeasy Plant Mini kit (Qiagen, Valencia, CA), including an on-column DNA digestion step.

The library preparation, Illumina sequencing, *de novo* assembly, read mapping and significance statistics, which are described below, were performed by BaseClear BV, the Netherlands (www.baseclear.com). From the total RNA, mRNA was purified, fragmented and converted to double stranded cDNA using the Illumina TruSeq RNA-Seq library preparation kit. Paired-end reads were obtained after 50 cycles on the TruSeq Illumina sequencer. Sequence reads were generated using the Illumina Casava pipeline version 1.8.2 and after passing quality assessments, filtered and trimmed reads, excluding duplicates, were assembled using the CLC Genomics Workbench version 5.1.0. Clean reads, including duplicates, from each sample were mapped individually to the consensus transcriptome and expression values for contigs from each sample were calculated using the CLC Genomics Workbench. A read consists of intact paired-end read pairs and single reads which, when mapping uniquely, are both counted as one hit. Broken pairs, where one partner is missing, were omitted from the quantification. Expression of each contig is displayed through an RPKM value for each petal type, which is the mean expression from the two technical replicates. Significant expression of each contig was evaluated performing a Baggerley’s test [74], followed by a Bonferroni correction for multiplicative errors.

### Data analysis and global gene expression comparisons

All contig sequences were blasted against an *Arabidopsis* protein database (TAIR10_pep_20110103_representative_gene_model) using BLASTX. Multiple contigs that retrieved the same *Arabidopsis* AGI-code were treated as partial transcripts of the same gene. Consequently, respective expression values from contigs with the same AGI code were totalled. Differentially expressed genes were defined as such when they had a p-value for statistical significance below 0.01 and had an expression fold change equal to or above two.

Functional annotation of *Iberis* sequences is based on the assignment of each contig to its respective *Arabidopsis* ortholog via a BLAST-search. For all downstream analyses only contig/AGI code matches that are supported with an e-value equal to or below e^−4^ were considered. Functional classification was performed using the classification superviewer tool at bar.utoronto.ca [36], based on MapMan annotations (file Ath_AGI_LOCUS_TAIR10_Aug2012.txt). Only categories with a p-value below 0.05 and with more than three members are listed. Note that in Figure 2 several MapMan categories are combined.

A statistical analysis of GO term enrichment (p-value <0.05) was done using FatiGO [38] against the 15,983 unigenes of the *Iberis* transcriptome (Table 4C) or against the *Arabidopsis* inflorescence transcriptome downloaded from TAIR (Table 5).

The *Arabidopsis* stage 15 petal and inflorescence transcriptomic data originate from a microarray analysis (TAIR-ME00319; AtGenExpress: Expression Atlas of *Arabidopsis* Development) and were downloaded from TAIR (accessions numbers: HybData:1007126238–40 and HybData:1007126271–73). Only elements that were present in all three replicates were considered as being expressed, AGI codes appearing more than once were removed.

### Quantitative real time PCR validation

Total RNA was isolated using the RNeasy Plant Mini kit (Qiagen, Valencia, CA), including an on-column DNA digestion step. Quantitative reverse transcriptase PCR was performed as described in [12]. Primers were designed online with the program Primer3 [75] and primer sequences are given in Additional file 8.

Specificity of each PCR was assessed by gel analysis and examination of the melt curve, generated after each reaction. The mean normalized expression (MNE) for each sample (calculated as in [12]) is based on three technical repetitions of each reaction, except for contigs 5537, 12149, 16003, 6640 and 7844 (Additional file 1 and 3), where for one single sample only two technical replicates were used. Error bars are standard error from technical replicates. The *Iberis Ran3 GTPase* [GenBank: EU145777] was used as a housekeeping gene for normalization.

Real time PCR was performed on adaxial and abaxial petals from stage A1 *Iberis* flowers on three biological replicates (replicates 5–7 in Figure 4D-I and Additional file 4A). The expression of *Arabidopsis* orthologs of *Iberis* genes to be tested was analysed using the *Arabidopsis* eFP browser at bar.utoronto.ca [76]. In cases, where orthologs were strongly expressed in pollen, two biological replicates of pollen-free adaxial and abaxial petals from stage A1 flowers were used (replicates 3, 4 in Figure 4A-C). Expression differences of *Arabidopsis* orthologs of *Iberis* genes assigned to the functional GO-categories connected to *cell wall* and *photosynthesis* were verified on two biological replicates of (pollen-free) adaxial and abaxial petals of stage A1 *Iberis* flowers, which were dissected into the upper petal blade and the lower petal claw (replicates 1, 2 in Figure 3 and Additional file 4B and C).

For the validation of gene expression differences detected with the RNA-Seq approach, qPCR was performed on stage A1 petal pairs of at least two biological replicates. A pair wise expression comparison of a given gene was performed and the expression value of abaxial petals (or petal claws, in cases of blade/claw comparison) was set to 1. An expression increase in adaxial petals of 1.5-times or above in at least two biological replicates was considered as a differential expression and consequently as a further corroboration of RNA-Seq data.

### Microarray analysis

Two technical samples of total RNA were prepared from each transgenic T2 Line expressing *IaTCP1*, *TCP1* and *CYC*, respectively, under the control of the *CaMV35S*-promoter [10] or the empty vector pBAR35S [GenBank: AJ251014] as a reference. Total RNA was extracted from plants showing clear flower phenotypes in the main inflorescence, harvesting secondary inflorescences that carried only young, closed flower buds. RNA isolation was conducted using the RNA isolation kit NucleoSpin RNA XS (Macherey and Nagel). Probe preparation, hybridization to ATH1 *Arabidopsis* Genome Arrays (Affymetrix, Santa Clara, California) and statistical data analysis were carried out at the Integrated Functional Genomic service unit from the University of Münster (Germany). Only genes showing a minimal expression fold change of two and a p-value ≤0.05 were considered as being differentially expressed. Microarray expression data were determined in comparison to transgenic plants harbouring the empty vector. Data were corroborated for selected genes by reverse transcriptase PCR performed as in [10] (Additional file 9), using the same plant material as for microarray analysis. Values for transcript accumulation derive from three PCR repetitions with an annealing temperature of 58°C, normalized to the expression strength of *Ran3 GTPase* (At5g55190). Primer sequences are given in Additional file 8, microarray data is given in Additional files 6, 10, 7.

### *In situ* hybridization

For *in situ* hybridization experiments an *IaH4* [GenBank: EU145778] antisense probe was hybridized to longitudinal sections of *I. amara* flower buds before anthesis, carried out as described in [10].

### Image aquisition

For scanning electronic microscopy, petals were frozen in liquid nitrogen, sputter coated with palladium in a cryo-preparation system (K1250X, Emitech) and examined with a digital scanning electron microscope (Zeiss Auriga). Stage A1 and A2 petals pictures were taken with a binocular with integrated camera (Leica M165 FC, Wetzlar).

### Availability of supporting data

The sequence datasets supporting the results of this article are available from the Short Read Archive (SRA) database at NCBI under the accession SRP048782. Assembled contigs are provided in FASTA format in Additional file 11. The microarray data have been deposited in the Gene Expression Omnibus (GEO) database at NCBI and are accessible under the accession GSE62213.

## Additional files

Additional file 1
**List of all 1,600** ***Iberis*** **genes with a significant expression in** ***Iberis*** **stage A1 adaxial and abaxial petals.** The list comprises all *Iberis amara* transcripts with a significant expression in petals, i.e. with a p-value for multiplicative error below 0.01 given in the colum “Bonferroni test”. All *Iberis* genes, represented by respective contig numbers, are assigned to AGI codes, based on a blast search against an *Arabidopsis* protein database. E-values obtained from that search are listed in the column “BLAST hit”. Only genes with an e-value cutoff of e^−4^ are listed. Each *Iberis*-gene is described by the annotation and MapMan classification of its respective AGI code. The expression of each gene in adaxial (aD) and abaxial (aB) petals is described with RPKM values (reads per kilobase per million of mapped reads) and summarized as fold change. Additional file 2
**List of 5% most highly expressed genes in abaxial and adaxial** ***Iberis amara*** **stage A1 petals.** As a subset of Additional file 1, this table lists all *Iberis amara* petal transcripts, which are amongst the 5% of most abundantly expressed genes in abaxial and adaxial stage A1 petals. Each *Iberis* transcript is represented by its corresponding AGI code, Annotation and MapMan bin. In addition, respective RPKM and fold change values are given. Additional file 3
**Genes differentially expressed between adaxial and abaxial stage A1** ***Iberis*** **petals.** As a subset of Additional file 1, this list contains all 334 transcripts, represented by respective contig numbers, with a minimal expression fold change of two between the two petal types. For each *Iberis* contig, an AGI code with corresponding annotation and MapMan characterization are given. Gene expression in adaxial (aD) and abaxial (aB) petals is described with RPKM and fold-change values. “Bonferroni test” and “BLAST hit” list p-values for multiplicative error and e-values resulting from a blast search of *Iberis* contigs against an *Arabidopsis* protein database, respectively. Additional file 4
**Photosynthesis-related genes do not contribute to monosymmetry formation.** A phosphoribulokinase was chosen as a representative for photosynthesis-related processes for validation of RNA-Seq data. (A) Expression in adaxial petals was compared to that in stage A1 abaxial petals from three independent biological samples (replicates 5–7). (B, C) Adaxial and abaxial petals from two biological replicates (replicates 1 and 2) were dissected into the upper petal blade and the lower petal claw. (B) The expression of the phosphoribulokinase in the blade and in the claw was compared between the adaxial and abaxial petals. (C) The abundance of the phosphoribulokinase transcript was compared between the petal blade and claw of the two petal types. Error bars are standard error from three technical replicates. aBp, abaxial petal; aBpb, abaxial petal blade; aBpc, abaxial petal claw; aDp, adaxial petal; aDpb, adaxial petal blade; aDpc, adaxial petal claw. Additional file 5
**Common putative target genes of** ***IaTCP1*** **in** ***Iberis amara*** **petals and of ectopically expressed** ***IaTCP1, TCP1*** **and** ***CYC*** **in** ***Arabidopsis*** **inflorescences.** Listed are genes that were found to be differentially expressed (≥2-fold) between adaxial and abaxial stage A1 petals of *Iberis amara* and in plants expressing either *IaTCP1, TCP1* or *CYC* under the control of the *35S*-promoter in *Arabidopsis thaliana* compared to plants carrying an empty vector. A – or + indicates activation or repression. Bold print highlights genes that are related to cell wall processes. Additional file 6
**List of 600 genes differentially regulated upon ectopic expression of** ***IaTCP1*** **in transgenic** ***Arabidopsis*** **.** Differentially expressed genes result from a microarray analysis of transgenic *Arabidopsis* inflorescences carrying young flower buds, which ectopically express *IaTCP1* under the control of the *35S-*promoter. Differential expression was deduced in comparison to transgenic plants harbouring an empty vector. All 600 genes show a minimal expression fold-change of two and a p-value equal to or below 0.05. For each probe set identifier, respective AGI codes, Annotation, MapMan bins and fold-change values (red) are given. In addition, respective values of fold-change (and p-values) from hybridization analyses with inflorescence material from transgenic plants overexpressing *TCP1* and *CYC* are listed. Additional file 7
**List of 328 genes differentially regulated upon ectopic expression of** ***TCP1*** **in transgenic** ***Arabidopsis*** **.** Differentially expressed genes result from a microarray analysis of transgenic *Arabidopsis* inflorescences carrying young flower buds, which ectopically express *TCP1* under the control of the *35S-*promoter. Differential expression was deduced in comparison to transgenic plants harbouring an empty vector. All 328 genes show a minimal expression fold-change of two and a p-value equal to or below 0.05. For each probe set identifier, respective AGI codes, Annotation, MapMan bins and fold-change values (blue) are given. In addition, respective values of fold-change (and p-values) from hybridization analyses with inflorescence material from transgenic plants overexpressing *IaTCP1* and *CYC* are listed. Additional file 8
**Primer list.** List of primers used to confirm RNA-Seq data in *Iberis amara* and microarray expression data in *Arabidopsis thaliana*. Additional file 9
**Confirmation of microarray expression data.** Microarray expression data of 28 genes differentially expressed due to ectopic activity of *IaTCP1*, *TCP1* or *CYC* in *Arabidopsis* inflorescences. Microarray expression data was confirmed by semi-quantitative RT PCR with three biological replicates from transgenic plants overexpressing *IaTCP1* or *CYC*. A minimal expression fold change of ≥2 in all three replicates is indicated with a +. Additional file 10
**List of 564 genes differentially regulated upon ectopic expression of** ***CYC*** **in transgenic** ***Arabidopsis*** **.** Differentially expressed genes result from a microarray analysis of transgenic *Arabidopsis* inflorescences carrying young flower buds, which ectopically express *CYC* under the control of the *35S-*promoter. Differential expression was deduced in comparison to transgenic plants harbouring an empty vector. All 564 genes show a minimal expression fold-change of two and a p-value equal to or below 0.05. For each probe set identifier, respective AGI codes, Annotation, MapMan bins and fold-change values (green) are given. In addition, respective values of fold-change (and p-values) from hybridization analyses with inflorescence material from transgenic plants overexpressing *IaTCP1* and *TCP1* are listed. Additional file 11
**The** ***Iberis amara*** **stage A1 petal transcriptome.** This list contains all *de novo* assembled 52,081 *Iberis amara* stage A1 petal sequences and respective contig numbers.
