# Supplementary figures and images for: Differential transcriptome analysis reveals insight into monosymmetric corolla development of the crucifer Iberis amara
Source: BMC Plant Biol. 2014 Nov 19;14:285. doi: 10.1186/s12870-014-0285-4 (PMC4245847; doi:10.1186/s12870-014-0285-4)

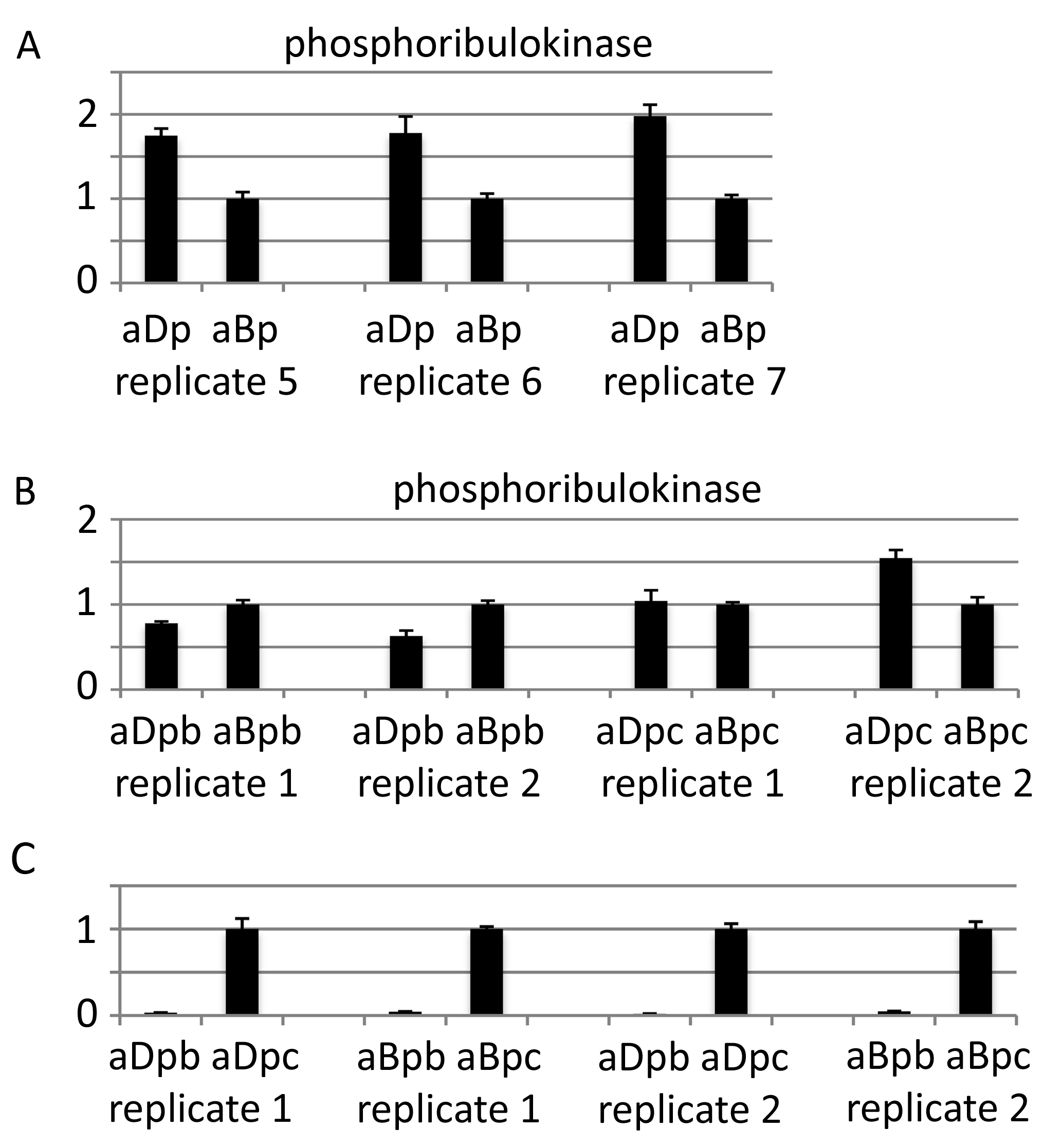

Supplement: Additional file 4 — Photosynthesis-related genes do not contribute to monosymmetry formation. A phosphoribulokinase was chosen as a representative for photosynthesis-related processes for validation of RNA-Seq data. (A) Expression in adaxial petals was compared to that in stage A1 abaxial petals from three independent biological samples (replicates 5–7). (B, C) Adaxial and abaxial petals from two biological replicates (replicates 1 and 2) were dissected into the upper petal blade and the lower petal claw. (B) The expression of the phosphoribulokinase in the blade and in the claw was compared between the adaxial and abaxial petals. (C) The abundance of the phosphoribulokinase transcript was compared between the petal blade and claw of the two petal types. Error bars are standard error from three technical replicates. aBp, abaxial petal; aBpb, abaxial petal blade; aBpc, abaxial petal claw; aDp, adaxial petal; aDpb, adaxial petal blade; aDpc, adaxial petal claw. [file 12870_2014_285_MOESM4_ESM.tiff]
