# Supplementary material for: Differential transcriptome analysis reveals insight into monosymmetric corolla development of the crucifer Iberis amara
Source: BMC Plant Biol. 2014 Nov 19;14:285. doi: 10.1186/s12870-014-0285-4 (PMC4245847; doi:10.1186/s12870-014-0285-4)
Supplement: Additional file 5 — Common putative target genes of IaTCP1 in Iberis amara petals and of ectopically expressed IaTCP1, TCP1 and CYC in Arabidopsis inflorescences. Listed are genes that were found to be differentially expressed (≥2-fold) between adaxial and abaxial stage A1 petals of Iberis amara and in plants expressing either IaTCP1, TCP1 or CYC under the control of the 35S-promoter in Arabidopsis thaliana compared to plants carrying an empty vector. A – or + indicates activation or repression. Bold print highlights genes that are related to cell wall processes. [file 12870_2014_285_MOESM5_ESM.pdf]

Target genes of	AGI Code	IaTCP1	TCP1	CYC	aDaxial petals (*I. amara*)	Annotation
IaTCP1	AT1G02790	-	0	0	5,04	**polygalacturonase 4**
IaTCP1	AT2G26450	-	0	0	5,69	**Plant invertase/pectin methylesterase inhibitor superfamily**
IaTCP1	AT3G62170	-	0	0	3,70	**VANGUARD 1 homolog 2**
IaTCP1	AT5G07430	-	0	0	4,98	**Pectin lyase-like superfamily protein**
IaTCP1	AT1G54070	-	0	0	4,77	Dormancy/auxin associated family protein
IaTCP1	AT4G27440	-	0	0	2,23	protochlorophyllide oxidoreductase B
IaTCP1	AT5G47000	-	0	0	4,85	Peroxidase superfamily protein
IaTCP1	AT3G20530	-	0	0	4,04	Protein kinase superfamily protein
IaTCP1	AT5G16500	-	0	0	4,59	Protein kinase superfamily protein
IaTCP1	AT2G43230	-	0	0	5,15	Protein kinase superfamily protein
IaTCP1	AT4G03290	-	0	0	4,62	EF hand calcium-binding protein family
IaTCP1	AT3G25170	-	0	0	4,77	ralf-like 26
IaTCP1	AT5G50790	+	0	0	8,40	Nodulin MtN3 family protein
IaTCP1	AT1G73220	-	0	0	4,26	organic cation/carnitine transporter1
IaTCP1	AT4G35180	-	0	0	3,53	LYS/HIS transporter 7
IaTCP1	AT2G26490	-	0	0	19,89	Transducin/WD40 repeat-like superfamily protein
IaTCP1	AT5G14890	-	0	0	4,17	NHL domain-containing protein
IaTCP1	AT1G25240	-	0	0	6,70	ENTH/VHS/GAT family protein
IaTCP1	AT1G02070	-	0	0	4,37	protein coding
IaTCP1	AT1G19500	-	0	0	4,24	protein coding, 0
IaTCP1	AT1G47280	-	0	0	3,28	no annotation
IaTCP1	AT1G63060	-	0	0	4,17	protein coding, 0
IaTCP1	AT4G28280	-	0	0	7,26	LORELEI-like-GPI anchored protein 3
CYC	AT5G61420	0	0	-	2,35	myb domain protein 28
CYC	AT4G32980	0	0	-	2,67	homeobox gene 1
CYC	AT4G14550	0	0	+	2,24	indole-3-acetic acid inducible 14
CYC	AT5G06530	0	0	+	20,83	Arabidospsi thaliana ATP-binding cassette G22
IaTCP1 TCP1	AT2G24450	-	-	0	4,79	FASCICLIN-like arabinogalactan protein 3 precursor
IaTCP1 TCP1	AT3G62710	-	-	0	2,81	Glycosyl hydrolase family protein
IaTCP1 TCP1	AT3G07820	-	-	0	5,02	**Pectin lyase-like superfamily protein**
IaTCP1 TCP1	AT5G15110	-	-	0	7,15	**Pectate lyase family protein**
IaTCP1 TCP1	AT3G17060	-	-	0	5,51	**Pectin lyase-like superfamily protein**
IaTCP1 TCP1	AT2G36020	-	-	0	4,92	HVA22-like protein J
IaTCP1 TCP1	AT1G29140	-	-	0	3,36	Pollen Ole e 1 allergen and extensin family protein
IaTCP1 TCP1	AT5G45880	-	-	0	5,01	Pollen Ole e 1 allergen and extensin family protein
IaTCP1 TCP1	AT5G19580	-	-	0	5,54	glyoxal oxidase-related protein
IaTCP1 TCP1	AT1G10770	-	-	0	4,99	**Plant invertase/pectin methylesterase inhibitor superfamily**
IaTCP1 TCP1	AT3G62180	-	-	0	6,17	**Plant invertase/pectin methylesterase inhibitor superfamily**
IaTCP1 TCP1	AT4G02250	-	-	0	4,02	**Plant invertase/pectin methylesterase inhibitor superfamily**
IaTCP1 TCP1	AT4G36490	-	-	0	2,21	ATSFH12, SEC14-like 12
IaTCP1 TCP1	AT3G61160	-	-	0	4,02	Protein kinase superfamily protein
IaTCP1 TCP1	AT2G26410	-	-	0	4,86	IQ domain 4
IaTCP1 TCP1	AT2G31500	-	-	0	2,02	calcium-dependent protein kinase 24
IaTCP1 TCP1	AT4G00350	-	-	0	5,37	MATE efflux family protein
IaTCP1 TCP1	AT3G17980	-	-	0	4,69	Calcium-dependent lipid-binding (CaLB domain) family
IaTCP1 TCP1	AT1G68110	-	-	0	2,02	ENTH/ANTH/VHS superfamily protein
IaTCP1 TCP1	AT1G13970	-	-	0	2,64	Protein of unknown function (DUF1336)
IaTCP1 TCP1	AT1G79910	-	-	0	3,40	Regulator of Vps4 activity in the MVB pathway protein
IaTCP1 TCP1	AT2G27180	-	-	0	5,13	protein coding
IaTCP1 TCP1	AT3G60780	-	-	0	14,01	Protein of unknown function (DUF1442)
IaTCP1 TCP1	AT5G39880	-	-	0	6,19	unknown function
IaTCP1 TCP1	AT5G50830	-	-	0	4,98	protein coding
IaTCP1 TCP1	AT5G04180	-	-	0	5,76	alpha carbonic anhydrase 3
IaTCP1 TCP1 CYC	AT3G11480	+	+	+	3,33	S-adenosyl-L-methionine-dependent methyltransferase
IaTCP1 TCP1 CYC	AT1G29660	+	+	+	2,06	GDSL-like Lipase/Acylhydrolase superfamily protein

IaTCP1 TCP1 CYC	AT4G19690	-	-	-	2,40	iron-regulated transporter 1
IaTCP1 TCP1 CYC	AT4G19380	-	-	-	2,52	Long-chain fatty alcohol dehydrogenase family protein
IaTCP1 TCP1 CYC	AT5G42680	+	+	+	3,61	Protein of unknown function, DUF617
IaTCP1 CYC	AT1G77760	-	0	-	2,50	nitrate reductase 1
IaTCP1 CYC	AT1G62770	-	0	-	4,25	**Plant invertase/pectin methylesterase inhibitor superfamily**
