# Supplementary material for: Differential transcriptome analysis reveals insight into monosymmetric corolla development of the crucifer Iberis amara
Source: BMC Plant Biol. 2014 Nov 19;14:285. doi: 10.1186/s12870-014-0285-4 (PMC4245847; doi:10.1186/s12870-014-0285-4)
Supplement: Additional file 8 — Primer list. List of primers used to confirm RNA-Seq data in Iberis amara and microarray expression data in Arabidopsis thaliana. [file 12870_2014_285_MOESM8_ESM.pdf]

Accession	Sequence 5’-3’	Gene
	qPCR for confirmation of RNAseq data	*Iberis amara*
EU145777 fwd	CTGTGGCAAGATTCGGTTTT	IaRAN3 (At5g55190)
EU145777 rev	TACCTGCTTGGCCTTCACTT	IaRAN3 (At5g55190)
EU145779 fwd	GGATCCACCAAAGGGGTCTA	IaTCP1 (At1G67260)
EU145779 fwd	GCTGCTACAACCATGATCCA	IaTCP1 (At1G67260)
Contig8362 fwd	AAACCATCGTGATCGGACTC	Phosphoribulokinase (At1G32060)
Contig8362 rev	ATTAGCACGTGGGTCCAAAG	Phosphoribulokinase (At1G32060)
Contig20004 fwd	AGCCATGTCAAAAGCGTAGC	Pectate lyase family (At5G15110)
Contig20004 rev	GGAATCAGACCGGCAATACA	Pectate lyase family (At5G15110)
Contig17630 fwd	CCGAAAGAAGAAGATGTTAGTGGT	Polygalacturonase 4 (At1G02790)
Contig17630 rev	CATCCACAACTTTACTTGGATCA	Polygalacturonase 4 (At1G02790)
Contig12149 fwd	GGTTCTGCTTTGGTCAATCC	Invertase/pectinmethylesterase (At2G26450)
Contig12149 rev	TCCTCTTTTGCGTCTTCCAC	Invertase/pectinmethylesterase (At2G26450)
Contig16003 fwd	TGAGAATGTAAAAGGTAACGTGGT	VGD 1 homolog 2 (At3G62170)
Contig16003 rev	CAAACTGTCATCCCCAAAAA	VGD 1 homolog 2 (At3G62170)
Contig5537 fwd	GATGGGAAACTCGCAAATGT	Glycosyl hydrolase 9A4 (At3G43860)
Contig5537 rev	GAGTCGATTCCTGCGAGAAG	Glycosyl hydrolase 9A4 (At3G43860)
Contig16048 fwd	TCATCAAAACTCTATGAACATTACGTC	Petal loss (At5G03680)
Contig16048 rev	GAATTACCATCATTCCCTTCTGA	Petal loss (At5G03680)
Contig3540 fwd	CCTCAAGAGATTATATTCAGGGAAA	Rcd1-like (At5G12980)
Contig3540 rev	TCACTTATTTCAATTGCAACATCC	Rcd1-like (At5G12980)
Contig7820 fwd	CATGAGAGCTCGATTCGACA	Unknown protein (At5G42680)
Contig7820 rev	CCGTAATAAGGACGTCAAGAAGA	Unknown protein (At5G42680)
Contig30658 fwd	CTCCTCCTCCGTTCAATCAG	Cytokinin oxidase 3 (At5G56970)
Contig30658 rev	CCACAACTCCGTCTTTAGCC	Cytokinin oxidase 3 (At5G56970)
Contig11132 fwd	TCATCGCTTTTATGACAGTA	P-glycoprotein 13 (At1G27940)
Contig11132 rev	CACTCTTTTTTGAGATTGTG	P-glycoprotein 13 (At1G27940)
Contig18979 fwd	CGATAAGGACACCGGAAAGT	COBL10 (At3G20580)
Contig18979 rev	ATGTTGAGGTGCGTGTAGAG	COBL10 (At3G20580)
Contig7844 fwd	GCACAACACACATGACATCG	RALF15 (At2G22055)
Contig7844 rev	GATGTGTTAAACATTTGGGAAAAA	RALF15 (At2G22055)
Contig6640 fwd	AGAAAAAAACACACAAATGGGT	RALF19 (At2G33775)
Contig6640 rev	AACACAAGATTTAGTCAATGTCCAC	RALF19 (At2G33775)
	Semiquantitative RT PCR for confirmation of microarray data	*Arabidopsis thaliana*
At5G55190 fwd	ACCAGCAAACCGTGGATTACCCTAGC	AtRAN3
At5G55190 rev	ATTCCACAAAGTGAAGATTAGCGTCC	AtRAN3
At3G66656 fwd	GTGAAACATCAGGCTATAGTAGAGG	AGL91
At3G66656 rev	GCTTATTTTCTTAATCTTAGC	AGL91
At1G01010 fwd	GGAGGATCAAGTTGGGTTTGG	ANAC001
At1G01010 rev	GGAGATGAACAACAAGACACC	ANAC001
At1G73830 fwd	GCAACACGCTTCATCATTTCC	BEE3
At1G73830 rev	CCATTTATGGAAACTTGAGC	BEE3
At2G26150 fwd	GCTTCTTCATCTGTAGGATCC	HSFA2
At2G26150 rev	GGTTCCGAACCAAGAAAACC	HSFA2
At1G74660 fwd	GCAGAGATCAAGAAACTCC	MIF1
At1G74660 rev	CGTTAGGTGGAGAATACTCAC	MIF1
At5G61590 fwd	CAACAACGTCACCTGAGG	AP2-domain protein
At5G61590 rev	CCTTGAGGCTGTGGTACATCG	AP2-domain protein
At5G61420 fwd	CCAAGGTCTCCGAGCTTAGC	MYB28
At5G61420 rev	CCTCATTGTGGTTATCTCCTCC	MYB28
At5G06710 fwd	CCACCATTACAGCTTCAGCTTCACTTCC	HAT14
At5G06710 rev	GCTGTCTTCAAGAAAAGCAGATTGG	HAT14
At1G69560 fwd	CCACTCATTAACCCTAATCC	MYB105
At1G69560 rev	GGTTTGGTTTAAGAAATCG	MYB105
At1G35490 fwd	GGAAATTTCGGATTCATTGG	bZIP
At1G35490 rev	GGTTGATATTTGTTGTATCG	bZIP
At3G06490 fwd	GGATTCAGTCAGCCTCGGCCTCATCC	MYB108
At3G06490 rev	CCATTTGACCCGAATAGTATTCC	MYB108
At2G25890 fwd	CCAATAATGAGGAGTCTCC	Glycin rich protein
At2G25890 rev	GCTCCCATCGGGTGTTTTCC	Glycin rich protein
At2G18660 fwd	CCAATCGCTGAAGCTGCTCAAGG	EXBL3
At2G18660 rev	GTGTATACGACACGAATGTTACC	EXBL3
At4G14365 fwd	CCATGAGGCAGAGAAGAGAAAGC	Zinc finger
At4G14365 rev	GACATGGCGACACGAGTCAAAGC	Zinc finger
At1G26800 fwd	CCAAGGCTTCCATCGATGCG	Zinc finger
At1G26800 rev	CCATCACTGTTTCCACCGTCC	Zinc finger
At1G13260 fwd	CGTTGTTAACGTCGGGGTTGAGTAATGATGG	RAV1
At1G13260 rev	CGTTTCTTGAACTCTCCGGTGAAATGTTAACTC	RAV1

At1G66230 fwd	CCTTTTCAAGAAACAATGGAGCGTCC	MYB20
At1G66230 rev	GTTCTCTATAATGTTGTCCACACC	MYB20
At4G23140 fwd	CCTTAAATCTTTGTCCCAAGG	CRK6
At4G23140 rev	CCAACGACTCCCTCGGTGGTGGTGG	CRK6
At4G26150 fwd	GGAAAGACAGAATGGTTATAACAACG	CGA1
At4G26150 rev	GCTCTGGGTCTCCAAATCCTCG	CGA1
At5G66815 fwd	GGTCAAAAGAAAACATTGTACG	Unknown protein
At5G66815 rev	GGATAAACTATGGCCAATGC	Unknown protein
At1G20190 fwd	CTCTAGCTGGATTGGCGGTTTTGGC	EXLB11
At1G20190 rev	CCGATCTTTTCCCAAGCGGG	EXLB11
At3G07830 fwd	GGACATGAACAGGACGTCAGC	Putative polygalacturonase
At3G07830 rev	GGGTCAGCACGGGTGGTGTGT	Putative polygalacturonase
At1G70720 fwd	CCACCTTTCTCCCACAAATCC	invertase/pectin methylesterase inhibitor
At1G70720 rev	CGTCATCTTTGATAGAGTCG	invertase/pectin methylesterase inhibitor
At1G29140 fwd	CCCTCGTTCACTTCACCGCA	Pollen Ole e 1 allergen and extensin
At1G29140 rev	GGCATTCAGCGAGAGGAGTCTTCC	Pollen Ole e 1 allergen and extensin
At3G62180 fwd	GGCTAAACCAGTCAAACGAACG	invertase/pectin methylesterase inhibitor
At3G62180 rev	GTAATAATAATCAGACAAGGGTG	invertase/pectin methylesterase inhibitor
At5G07430 fwd	CAATGTCCGTGGTTGCATTCC	Pectin lyase like superfamily protein
At5G07430 rev	ATCCGCGGTTGAGGTTCTCCC	Pectin lyase like superfamily protein
At5G56870 fwd	ATGGTATTGAACTTTAGAG	BGAL4
At5G56870 rev	CGCTGCAGAGTTCTCATCTTTGTTC	BGAL4
At3G17060 fwd	CGCATCAGCAATGCAAAAGAC	Pectin lyase like superfamily protein
At3G17060 rev	GGGTCGAGCCATGGTAACTCC	Pectin lyase like superfamily protein
At1G47960 fwd	GAGACACCAGACTTCAATC	VIF1, pectinesterase
At1G47960 rev	GTTGCTTCGACACCGGCGTC	VIF1, pectinesterase
At4G30140 fwd	GGTGGCACCAGCGGTGCATGG	GDSL-motif lipase
At4G30140 rev	GAACATAGCTCCCGGTTTGTTG	GDSL-motif lipase
At3G50660 fwd	CGCTTAACTTCTTAAGTCACGCACG	DWF4
At3G50660 rev	CGAGAAACCCTAATAGGCAAACC	DWF4
At2G16910 fwd	GGACAGAACTCATAGCGGAGAATGG	AMS
At2G16910 rev	GGTTGTGGTAATGGTTGATGTGTTGG	AMS
At4G08950 fwd	CCTACGTCCAAAACTCTCCACC	EXO
At4G08950 rev	GCACCATACGCATTAAAACTACC	EXO
At2G34870 fwd	GCTTCTTCATCACTCATCAGTGTAGC	MEE26
At2G34870 rev	GGAACACCGCCAAATGG	MEE26
At5G57560 fwd	GCGATCACTTACTTGCTTCC	XTH22_TCH4
At5G57560 rev	GCCAGTAGTAGTCCCCTGTTTCGAGGC	XTH22_TCH4
At1G68360 fwd	GGAACGTGGCGAGTTTCCGG	Zinc finger-like
At1G68360 rev	CGGCTGCGTTTCGTGTAGC	Zinc finger-like
At3G20450 fwd	CGTGACAATCGAAGCCGTCC	Unknown protein
At3G20450 rev	GGAAGCTTCGAGGAGG	Unknown proten
At3G63010 fwd	CGTCTCTCCTTCATCAGACCCGTCACG	ATGID 1B hydrolase
At3G63010 rev	GGAGTAAGAAGCACAGGACTTGACTTGC	ATGID 1B hydrolase
