# Supplementary material for: Differential transcriptome analysis reveals insight into monosymmetric corolla development of the crucifer Iberis amara
Source: BMC Plant Biol. 2014 Nov 19;14:285. doi: 10.1186/s12870-014-0285-4 (PMC4245847; doi:10.1186/s12870-014-0285-4)
Supplement: Additional file 9 — Confirmation of microarray expression data. Microarray expression data of 28 genes differentially expressed due to ectopic activity of IaTCP1, TCP1 or CYC in Arabidopsis inflorescences. Microarray expression data was confirmed by semi-quantitative RT PCR with three biological replicates from transgenic plants overexpressing IaTCP1 or CYC. A minimal expression fold change of ≥2 in all three replicates is indicated with a +. [file 12870_2014_285_MOESM9_ESM.pdf]

AGI Code	Annotation	Affymetrix fold change	sqRT-PCR
**IaTCP1/TCP1**			
At3g63010	ATGID1B (GA INSENSITIVE DWARF 1B); hydrolase	2,68 / 2,14	+
At3g06490	AtMYB108; transcription factor	-4,20 / -2,87	+
At1g35490	bZIP transcription factor family	-5,14 / -2,88	+
At1g70720	Plant invertase/pectin methylesterase inhibitor superfamily	-4,62 / -2,16	+
At3g07830	Putative polygalacturonase/putative pectinase	-3,58 / -2,12	+
At5g56870	BGAL4 (BETA- GALACTOSIDASE 4)	-3,10 / -2,60	+
At1g29140	Pollen Ole e 1 allergen and extensin family protein	-5,98 / -2,89	+
At3g17060	Pectin lyase like superfamily protein	-2,87 / -2,24	+
**IaTCP1**			
At2g18660	EXLB3 (EXPANSIN-LIKE B3 PRECURSOR)	6,70	+
At4g14365	Zinc finger (C3HC4-Typ) protein	4,62	+
At3g66656	AGL91; transcription factor	5,24	+
At4g26150	CGA1; CYTOKININ-RESPONSIVE GATA FACTOR 1; transcription factor	2,25	+
At2g25890	Glycine-rich protein/oleosin	-2,90	+
At1g73830	BEE3 (BR ENHANCED EXPRESSION 3); transcription factor	-2,81	+
At1g47960	VIf1; pectinesterase/pectinesterase inhibitor	-2,89	+
At1g20190	ATEXP11 (ARABIDOPSIS THALIANA EXPANSIN 11), expansin	-5,00	+
At4g30140	GDSL-motiv lipase/hydrolase	-2,98	+
**CYC**			
At1g74660	MIF1 (MINI ZINC FINGER 1); transcription factor	7,91	+
At5g61590	TF, protein family with AP2-domain	7,21	+
At1g66230	AtMYB20; transcription factor	2,72	+
At1g69560	AtMYB105; transcription factor	-2,46	+
At5g06710	HAT14 (HOMEOBOX of A. THALIANA); transcription factor	-2,46	+
At1g68360	Zinc finger-like protein	-2,43	+
At3g20450	Unknown protein	-3,93	+
At5g61420	AtMYB28; transcription factor	-4,23	+
At5g57560	XTH22_TCH4 (TOUCH 4); hydrolase	24,00	+
At2g16910	AMS (ABORTED MICROSPORES); transcription factor	-3,50	+
**IaTCP1/TCP1/CYC**			
At3g50660	DWF4 (DWARF 4); steroid 22-alpha hydroxylase	3,27 / 4,53 / 2,76	+ (+TCP1)
